# *N*-dodecanoyl-homoserine lactone influences the levels of thiol and proteins related to oxidation-reduction process in *Salmonella*

**DOI:** 10.1371/journal.pone.0204673

**Published:** 2018-10-10

**Authors:** Felipe Alves de Almeida, Deisy Guimarães Carneiro, Tiago Antônio de Oliveira Mendes, Edvaldo Barros, Uelinton Manoel Pinto, Leandro Licursi de Oliveira, Maria Cristina Dantas Vanetti

**Affiliations:** 1 Department of Microbiology, Universidade Federal de Viçosa, Viçosa, Minas Gerais, Brazil; 2 Department of Biochemistry and Molecular Biology, Universidade Federal de Viçosa, Viçosa, Minas Gerais, Brazil; 3 Núcleo de Análise de Biomoléculas, Universidade Federal de Viçosa, Viçosa, Minas Gerais, Brazil; 4 Food Research Center, Department of Food and Experimental Nutrition, Faculty of Pharmaceutical Sciences, Universidade de São Paulo, São Paulo, São Paulo, Brazil; 5 Department of General Biology, Universidade Federal de Viçosa, Viçosa, Minas Gerais, Brazil; Oswaldo Cruz Foundation, BRAZIL

## Abstract

Quorum sensing is a cell-cell communication mechanism mediated by chemical signals that leads to differential gene expression in response to high population density. *Salmonella* is unable to synthesize the autoinducer-1 (AI-1), *N*-acyl homoserine lactone (AHL), but is able to recognize AHLs produced by other microorganisms through SdiA protein. This study aimed to evaluate the fatty acid and protein profiles of *Salmonella enterica* serovar Enteritidis PT4 578 throughout time of cultivation in the presence of AHL. The presence of *N*-dodecanoyl-homoserine lactone (C12-HSL) altered the fatty acid and protein profiles of *Salmonella* cultivated during 4, 6, 7, 12 and 36 h in anaerobic condition. The profiles of *Salmonella* Enteritidis at logarithmic phase of growth (4 h of cultivation), in the presence of C12-HSL, were similar to those of cells at late stationary phase (36 h). In addition, there was less variation in both protein and fatty acid profiles along growth, suggesting that this quorum sensing signal anticipated a stationary phase response. The presence of C12-HSL increased the abundance of thiol related proteins such as Tpx, Q7CR42, Q8ZP25, YfgD, AhpC, NfsB, YdhD and TrxA, as well as the levels of free cellular thiol after 6 h of cultivation, suggesting that these cells have greater potential to resist oxidative stress. Additionally, the LuxS protein which synthesizes the AI-2 signaling molecule was differentially abundant in the presence of C12-HSL. The NfsB protein had its abundance increased in the presence of C12-HSL at all evaluated times, which is a suggestion that the cells may be susceptible to the action of nitrofurans or that AHLs present some toxicity. Overall, the presence of C12-HSL altered important pathways related to oxidative stress and stationary phase response in *Salmonella*.

## 1. Introduction

Quorum sensing is a mechanism of cell-cell communication involved in transcription regulation in response to signaling molecules that accumulate according to high cell density [1, 2]. Quorum sensing regulates a range of phenotypes in bacteria such as production of virulence factors, biofilm formation, protease production, swarming motility, pigment production, bioluminescence, among others [3]. In *Salmonella*, this mechanism can be mediated by three types of autoinducers (AI), denominated AI-1, AI-2 and AI-3 [4–8]. The signaling molecules known as AI-1 are *N*-acyl homoserine lactones (AHLs) [4, 6, 9, 10], the AI-2 molecules are (2*R*, 4*S*)-2-methyl-2,3,3,4-tetrahydroxytetrahydrofuran (*R*-THMF) derived from (*S*)-4,5-dihydroxy-2,3-pentanedione (DPD) [11, 12] and the AI-3 are molecules produced by the gastrointestinal microbiota or hormones produced by the host such as norepinephrine and epinephrine [6, 13–18]. In addition, indole is a cell-cell communication molecule used by several Gram-positive and Gram-negative bacteria playing important biological roles such as spore formation, drug resistance, virulence, plasmid stability, and biofilm formation [8, 19, 20]. However, high concentrations of indole seem to inhibit AHL mediated signaling in *Salmonella enterica* serovar Typhimurium [21]. In addition to the described molecules, other types of signaling cues have been described for different microbes in the literature such as quinolones, diketopiperazines, hydroxyketones, gamma-butyro-lactones, among others [3].

The cell-cell communication mechanism mediated by AI-1 in the Proteobacteria phylum is composed of a pair of proteins named LuxI (acyl homoserine lactone synthase) and LuxR (transcriptional factor) and its homologous proteins. In contrast, some Proteobacteria belonging to the Enterobacteriaceae family, such as *Salmonella* spp. and *Escherichia coli*, do not synthesize AHL since they lack a LuxI homologue [9, 10, 22]. However, a homologue of LuxR, known as SdiA, is present in these bacteria and allows the detection of AHLs synthesized by other microorganisms such as *Aeromonas hydrophila* and *Yersinia enterocolitica*, leading to gene regulation [23, 24]. These AIs are able to enter and leave the cell by diffusion or through efflux pumps depending on the type of AHL [1, 25–27]. The AHL molecules bind to the N-terminal domain of the SdiA protein, altering the binding affinity of its C-terminal domain to the DNA and, consequently, regulating the expression of target genes [2, 28–31].

It has been shown that AHLs regulate the expression of the *rck* operon (resistant to complement killing), which codes for *pefI*, *srgD*, *srgA*, *srgB*, *rck* and *srgC* genes, and it is found in plasmids influencing virulence of *Salmonella* Typhimurium [9, 10, 32, 33]. Campos-Galvão et al. [34] also showed that the *hilA*, *invA* and *invF* genes present in the Pathogenicity Islands 1 (SPI-1) of *S*. *enterica* serovar Enteritidis PT4, and *glgC*, *fliF*, *lpfA* and *fimF* genes, involved in biofilm formation, were up-regulated in the presence of *N*-dodecanoyl homoserine lactone (C12-AHL).

A global analysis was carried out on the influence of AHL on the abundance of proteins and the levels of extracellular organic acids of *Salmonella* Enteritidis [35]. It was shown that the abundance of proteins involved in translation (PheT), transport (PtsI), metabolic processes (TalB, PmgI, Eno and PykF) and response to stress (HtpG and Adi) increased while the abundance of other proteins related to translation (RplB, RplE, RpsB and Tsf), transport (OmpA, OmpC and OmpD) and metabolic processes (GapA) decreased at 7 h of cultivation in the presence of AI-1. Additionally, there were changes in the formate consumption. It was hypothesized that these observed changes are correlated with entry into the stationary phase of growth. In other organisms, the effect of AHLs in anticipating the stationary phase responses was confirmed by global analysis, such as the transcriptome of *Pseudomonas aeruginosa* [36] and *Burkholderia thailandensis* [37], as well as the metabolomes of *Burkholderia glumae*, *Burkholderia pseudomallei* and *B*. *thailandensis* [38].

Besides the global changes in intracellular metabolites described above, other phenotypes are altered when *Salmonella* is grown in the presence of AIs. For instance, Nesse et al. [39] showed that invasion of HEp-2 cells by *Salmonella* Typhimurium was increased in the presence of *N*-hexanoyl homoserine lactone (C6-HSL) and *N*-octanoyl homoserine lactone (C8-HSL) at 37 °C. The addition of C8-HSL in cells of *S*. *enterica* serovar Typhi containing plasmid pRST98, which harbors the virulence gene *rck*, increased adhesion to HeLa cells after 1 h at 37 °C in the presence of 5% CO_2_ gas [40]. Biofilm formation by *Salmonella* Enteritidis PT4 578 in polystyrene surface was positively regulated by C12-HSL after 36 h in anaerobiosis, even though no growth changes were observed in planktonic cells [34, 41]. Similarly, Bai and Rai [42] showed that biofilm formation on polystyrene was increased by *Salmonella* Typhimurium when cultivated with *N*-butyryl homoserine lactone (C4-HSL) e C6-HSL.

On the other hand, a cell free supernatant (CFS) rich in AHLs, AI-2 and other unknown compounds of *Y*. *enterocolitica* and *Serratia proteamaculans* altered growth of different phagetypes of *Salmonella* Enteritidis and *Salmonella* Typhimurium under aerobiosis [43]. Similarly, the CFS of *P*. *aeruginosa* containing AHLs and different metabolites decreased growth of nine serovars of *S*. *enterica* [44]. Conversely, the CFS of *Hafnia alvei* containing AHLs, as well as the addition of synthetic *N*-3-oxo-hexanoyl homoserine lactone (3-oxo-C6-HSL) to the growth medium in aerobiosis did not influence biofilm formation by *Salmonella* Typhimurium [45]. It is noteworthy that in these studies, the CFS of different bacteria contained metabolites other than AHLs which might have interfered in the detection of the AIs subtle effects in the cells.

According to Atkinson and Williams [29], the absence of an AHL synthase in *Salmonella* and *E*. *coli* can be related to ecological aspects, since the bacterium would avoid the information transfer to other microorganisms present in the medium and there would be no energy expenditure with the synthesis of these signaling molecules. However, these bacteria would be favored by the environmental information provided by others through the AHLs detection. Thus, what remains uncertain is the real advantage for *Salmonella* to use cell-cell communication mediated by AI-1. According to Di Cagno et al. [46], the global analyses of the proteome and transcriptome should help elucidate the influences of chemical signals on the cellular physiology. However, most studies on AHL signaling in *Salmonella* were targeted at specific genes, as previously mentioned [9, 10, 32, 34], while overall changes in *Salmonella* physiology in the presence of AHLs is still largely unknown.

The aim of this work was to evaluate the altered physiological aspects of *Salmonella*, such as the cells fatty acid and protein profiles during growth in the presence of AHL. This is the first work that carefully examines the AHL effect throughout growth in anaerobic conditions and the first to show that levels of thiol and proteins related to the oxidation-reduction stress response are altered by a quorum sensing signal in *Salmonella*, suggesting that these cells may have a greater potential to resist oxidative stress.

## 2. Materials and methods

### 2.1. Bacterial strain

*S*. *enterica* serovar Enteritidis phage type 4 (PT4) 578 (GenBank: MF066708.1), isolated from chicken meat, was provided by Fundação Oswaldo Cruz (FIOCRUZ, Rio de Janeiro, Brazil). Culture was stored at -20 °C in Luria-Bertani (LB) broth [47] supplemented with 20% (v/v) of sterilized glycerol.

### 2.2. Inoculum preparation

Inoculum was prepared according to Almeida et al. [35, 41]. Tryptone soy broth (TSB; Sigma-Aldrich, India) was prepared with CO_2_ under O_2_-free conditions, dispensed in anaerobic bottles that were sealed with butyl rubber stoppers and then autoclaved (anaerobic TSB). Before each experiment, cells were cultivated in 20 mL of anaerobic TSB for 24 h at 37 °C. Then, 1.0 mL was transferred into 10 mL of anaerobic TSB and incubated at 37 °C. After 4 h of incubation, exponentially growing cells were harvested by centrifugation at 5000 *g* at 4 °C for 10 min (Sorvall, USA), washed with 0.85% saline, and the pellet resuspended in 0.85% saline. The inoculum was standardized to 0.1 of optical density at 600 nm (OD_600nm_) (approximately 10^7^ CFU/mL) using a spectrophotometer (Thermo Fisher Scientific, Finland).

### 2.3. Preparation of HSL solution

The autoinducer-1, *N*-dodecanoyl-DL-homoserine lactone (C12-HSL; PubChem CID: 11565426; Fluka, Switzerland) was suspended in acetonitrile (PubChem CID: 6342; Merck, Germany) at a concentration of 10 mM and further diluted to a working solution of 10 μM in acetonitrile. Control experiment was performed using acetonitrile with final concentration in the media less than 1% (v/v) to avoid interference in the growth and response of *Salmonella* to C12-HSL [9].

### 2.4. Fatty acid profile analysis of *Salmonella*

#### 2.4.1. Saponification, methylation and extraction of fatty acids

An aliquot of 10 mL of the standardized inoculum was added into anaerobic bottles containing 90 mL of anaerobic TSB supplemented with 50 nM of C12-HSL or the equivalent volume of acetonitrile as control and then, incubated at 37 °C. After 4, 6, 7, 12 and 36 h of incubation, OD_600nm_ was determined. Concomitantly, 10 mL of the culture was centrifuged at 5000 *g* at 4 °C for 15 min (Sorvall, USA). The cells in the pellet were resuspended in 1 mL of sterilized distilled water, and once again, centrifuged at 5000 *g* at 4 °C for 10 min. The pellet was transferred to glass tubes free from fatty acids and afterwards, the fatty acids were saponified, methylated, and extracted by the procedure of the Sherlock^®^ Analysis Manual (version 6.2; MIDI, USA) [48].

#### 2.4.2. Analysis and identification of fatty acids

The cellular fatty acid composition was determined by 7890A gas chromatograph equipped with flame ionization detector (Agilent Technologies, USA). Afterwards, the fatty acids were identified and quantified in the Sherlock^®^ Microbial Identification System software by the procedure of the Sherlock^®^ Analysis Manual (version 6.2; MIDI, USA) using the Calibration Standard 1 (Microbial ID Part # 1200-A) containing the straight chain C9:0 to C20:0 fatty acid methyl esters [48].

#### 2.4.3. Statistical analysis

Experiments were carried out in three biological replicates. The values of the triplicates were used for Principal Component Analysis (PCA) using RStudio software (version 1.0.143; USA). All data were subjected to analysis of variance (ANOVA) followed by Tukey’s test using the Statistical Analysis System and Genetics Software^®^ [49]. A *p*-value < 0.05 was considered to be statistically significant.

### 2.5. Protein profile analysis of *Salmonella*

#### 2.5.1. Extraction and quantification of proteins

An aliquot of 10 mL of the standardized inoculum was added into anaerobic bottles containing 90 mL of anaerobic TSB supplemented with 50 nM of C12-HSL or the equivalent volume of acetonitrile as control and then, incubated at 37 °C. After 4, 6, 7, 12 and 36 h of incubation, OD_600nm_ was determined and 10 mL of the culture was centrifuged at 5000 *g* at 4 °C for 15 min (Sorvall, USA). The cells in the pellet were resuspended in 1 mL of sterilized distilled water, transferred to 1.5 mL microtubes and once again centrifuged at 9500 *g* at 4 °C for 30 min (Brikmann Instruments, Germany). The pellet was resuspended in 1 mL of Tris-HCl 50 mM, pH 8.0 added of 1 mM phenylmethylsulfonyl fluoride (PMSF) and 1 mM dithiothreitol (DTT). Next, the mixture was kept in ice for 1 min, then 1 min in ultrasound bath (154 W, 37 KHz; Unique, Brazil) and this cycle was repeated five times. Posteriorly, five cycles of 1 min in ice, 1 min in vortex and 8 min in ultrasound bath were performed. The mixture was centrifuged at 9500 *g* at 4 °C for 30 min, the supernatant containing the intracellular proteins was transferred to 1.5 mL microtube and stored at −20 °C. The pellet was resuspended in 100 μL of ammonium bicarbonate 50 mM and 1 mL of 2:1 trifluoroethanol:chloroform (TFE:CHCl_3_) was added. Then, the mixture was subjected again to five cycles on ice and ultrasound bath and five cycles on ice, vortex and ultrasound bath as previously described. The mixture was centrifuged at 9500 *g* at 4 °C for 4 min to obtain three phases. The upper phase, composed by proteins soluble in TFE, was transferred to 1.5 mL microtubes and was dried in SpeedVac (Genevac, England). The supernatant with intracellular proteins was transferred to microtube containing the proteins soluble in TFE. The protein extract was precipitated with 10% (w/v) trichloroacetic acid (TCA) and kept on ice for 30 min and after that, the material was centrifuged at 9500 *g* at 4 °C for 10 min. The supernatant was discarded and the precipitate washed three times with cold acetone. After evaporation of the residual acetone at room temperature, the precipitate was resuspended in 400 μL of ammonium bicarbonate 50 mM. Proteins were quantified using Coomassie blue dye [50] and the protein extracts were stored at -20 °C.

#### 2.5.2. In-solution protein digestion

The trypsin digestion of proteins in solution was performed according to Villen and Gygi [51], with modifications. An aliquot of the extract containing 10 μg of protein was transferred to 1.5 mL microtube and the final volume was adjusted to 150 μL with ammonium bicarbonate 50 mM and 150 μL of urea 8 M were added. The disulfide bonds of the proteins were reduced by 5 mM DTT for 25 min at 56 °C and the protein mixture was cooled to room temperature. Next, the alkylation was carried out by adding 14 mM iodoacetamide and followed by incubation for 30 min at room temperature in the dark. The free iodoacetamide was quenched by adding 5 mM DTT with further incubated for 15 min at room temperature in the dark. The urea concentration in the protein mixture was reduced to 1.6 M by diluting it in 1:5 in ammonium bicarbonate 50 mM with the addition of 1 mM calcium chloride. An aliquot containing 20 ng of trypsin (Sequencing grade modified trypsin; Promega, USA) in ammonium bicarbonate 50 mM was added to a final ratio of 1:50 of trypsin:protein and it was incubated for 16 h at 37 °C. After the incubation, the solution was cooled to room temperature and the enzymatic reaction was quenched with 1% (v/v) trifluoroacetic acid (TFA) until pH 2.0. After centrifugation at 2500 *g* for 10 min at room temperature, the supernatant containing the peptides was collected.

#### 2.5.3. Peptide desalting

The supernatant containing the peptides was desalted according to Rappsilber et al. [52], with modifications. Two membranes of octadecyl (C18; 3M EMPORE, USA) were packed in each stage tip to load 10 μg of digested proteins. The stage tip was conditioned with 100 μL of 100% (v/v) methanol and was equilibrated with 100 μL 0.1% (v/v) of formic acid. After that, the supernatant containing the peptides was loaded two times onto the stage tip and this was washed 10 times with 100 μL of 0.1% (v/v) of formic acid. The peptides were eluted with 200 μL of 80% (v/v) acetonitrile containing 0.1% (v/v) of formic acid. In each previous step, the stage tip was centrifuged at 400 *g* for 2 min (Eppendorf, Germany). The eluate was dried in SpeedVac (Thermo Fisher Scientific, Finland), resuspended in 22.5 μL of 0.1% (v/v) formic acid and stored at -20 °C.

#### 2.5.4. Mass spectrometric analysis

The solution containing the peptides was centrifuged at 9000 *g* for 5 min and 15 μL of the supernatant was transferred to vials. An aliquot of 4.5 μL of peptides, equivalent to 2 μg of protein, was separated by C18 (100 mm x 100 mm) RP-nanoUPLC (nanoAcquity; Waters, USA) coupled to a Q-Tof Premier mass spectrometer (Waters, USA) with nanoelectrospray source at a flow rate of 0.6 μL.min^-1^. The gradient was 2–90% (v/v) acetonitrile in 0.1% (v/v) formic acid over 45 min. The nanoelectrospray voltage was set to 3.0 kV, a cone voltage of 50 V and the source temperature was 80 °C. The instrument was operated in the ‘top three’ mode, in which one MS spectrum is acquired followed by MS/MS of the top three most-intense peaks detected. After MS/MS fragmentation, the ion was placed on exclusion list for 60 s.

#### 2.5.5. Identification and quantification of proteins

The spectra were acquired using software MassLynx (version 4.1; Waters) and the .*raw* data files were converted to a peak list format .*mgf* without summing the scans by the Mascot Distiller software (version 2.3.2.0; Matrix Science, United Kingdom) and searched against the knowledgebase UniProtKB using the Mascot software (version 2.4.0; Matrix Science, United Kingdom). For the search, the following parameters were considered: taxonomy *Salmonella* and all entries (separately), monoisotopic mass, trypsin, allowing up to one missed cleavage site, peptide charge +2, +3 and +4, fixed modification for carbamidomethylation of cysteine residues and variable modification for oxidation of methionine residues, peptide and MS/MS tolerance equal to 0.1 Da [53–55], ESI-QUA-TOF for instrument. The proteins identifications by Mascot software were validated and the proteins were quantified by Scaffold software (version 4.7.2; Proteome Software, USA). For the protein validation, peptide and protein identifications were accepted if they could be established at higher than 90% probability as specified by the Peptide Prophet algorithm [56] and by the Protein Prophet algorithm [57], respectively. In addition, the proteins should contain at least one identified peptide. The false discovery rates (FDR) for identification of proteins and peptides using decoy method by Scaffold software should to be less than or equal to 1% (FDR ≤ 1%) [54, 58, 59]. For the protein quantitation, the quantitative value of total ion current (TIC) of each protein was normalized by sum total of TIC. For the unidentified proteins, a TIC value of 0.05 was adopted (cut off of method sensibility).

#### 2.5.6. Statistical analysis

Experiments were carried out in three biological replicates. The logarithms of normalized TIC values of the triplicates were used for PCA as well as the logarithm of normalized mean of TIC values of the triplicates of each protein which were used to construct the heatmap and dendrogram using RStudio software. The statistical difference of normalized TIC values among the samples were calculated by T-test with correction for multiple hypotheses by FDR using RStudio software. The fold changed was calculated as the ratio of the normalized mean of TIC values of the treatment with C12-HSL by the control and the result was shown as logarithm in base two (Log_2_ FC). The proteins with *p*-value less than 0.05 (*p*-value < 0.05) or negative logarithm of *p*-value more than 1.301 (-Log_10_ *p* > 1.301) and fold changed less than 0.667-fold or more than 1.500-fold or Log_2_ FC less than -0.585 or more than 0.585 (Log_2_ FC < -0.585 or > 0.585) were considered differentially abundant proteins [60]. Moreover, when the protein was not detected in one of the treatments, the *p*-value was not considered and the fold-changed was calculated by adopting a TIC value of 0.05 (cut off of method sensibility) for the sample in which the protein was not identified, being this protein also considered differentially abundant.

#### 2.5.7. Bioinformatics analysis

The Gene Ontology (GO) annotations of the process and function of proteins were acquired with the tool QuickGO, implemented by the European Bioinformatics Institute (http://www.ebi.ac.uk/QuickGO/). Then, the Protein-Protein Interaction (PPI) network was generated for some proteins of *Salmonella* Typhimurium LT2 using the STRING database version 10.5 (http://string-db.org/, [61, 62]). The confidence levels of the PPI were considered in relation to the average local clustering coefficient: interactions at low confidence or better (coefficient ≥ 0.150), interactions at medium confidence or better (coefficient ≥ 0.400), interactions at high confidence or better (coefficient ≥ 0.700) and interactions at highest confidence (coefficient ≥ 0.900), available at http://string-db.org/ [61, 62].

### 2.6. Quantification of free cellular thiol

#### 2.6.1. Extraction of free cellular thiol

The pellet obtained as described in item 2.5.1. was resuspended in 250 μL of sterilized distilled water. Next, the mixture was kept on ice for 1 min, 1 min mixed by vortex for 1 min and treated with ultrasound (400 W, 20 KHz; Sonics & Materials Inc., USA) for 30 s; and this cycle was repeated five times. The mixture was then centrifuged at 9500 *g* at 4 °C for 15 min and the supernatant containing the free cellular thiol was used immediately.

#### 2.6.2. Quantification of free cellular thiol

The quantification of free cellular thiol was performed according to Ellman [63] and Riddles et al. [64], with modifications. For each 25 μL of the supernatant or standard, 5 μL of 0.4% (w/v) 5,5′-dithiobis(2-nitrobenzoic acid) (DTNB or Ellman’s reagent; Sigma, USA) in buffer sodium phosphate 0.1 M pH 8.0 containing 1mM EDTA and 250 μL of the buffer sodium phosphate were added in microplate. The microplate was incubated at room temperature for 15 min and the absorbance at 412 nm measured by using a spectrophotometer (Thermo Fisher Scientific, Finland). The free cellular thiol was quantified by using cysteine hydrochloride monohydrate (Sigma, USA) as standard at concentrations from 0.0 to 1.5 mM. The obtained equation was as follows: absorbance = (0.9421 x concentration) + 0.0432, with R^2^ = 0.9936. Next, the quantification of free cellular thiol was normalized by OD_600nm_.

#### 2.6.3. Statistical analysis

Experiments were carried out in four biological replicates. The statistical analysis was performed according to item 2.4.3, except that the PCA was not performed.

## 3. Results and discussion

### 3.1. HSL alters the fatty acid profile of *Salmonella* throughout time

Analysis of the fatty acid profile of *Salmonella* cells, both in the absence and presence of C12-HSL, showed alterations in the composition at different time points. The largest change appears to be at the 4 h time point with four fatty acids presenting altered abundance at this time of cultivation. Of these fatty acids, 17:0 cyclo ɷ7c and 19:0 cyclo ɷ8c had their abundance decreased in the presence of C12-HSL, while two unresolved mixtures of 16:1 ɷ6c/16:1 ɷ7c and 18:1 ɷ6c/18:1 ɷ7c had their abundance increased (Table 1 and S1 Table).

**Table 1:** Fatty acid profile of *Salmonella* Enteritidis PT4 578 anaerobically cultivated in TSB at 37 °C in the presence or absence of C12-HSL.

Classification	Fatty acids	Time (h)									
		4		6		7		12		36	
		Control	C12-HSL	Control	C12-HSL	Control	C12-HSL	Control	C12-HSL	Control	C12-HSL
Saturated	12:00	4.80^A^	4.72^A^	4.69^AB^	4.61^AB^	4.46^C^	4.65^AB^	4.31^C^	4.38^B^	4.50^BC^	4.50^AB^
	14:00	6.68^E^	6.85^E^	9.21^D^	9.21^D^	9.82^C^	10.21^C^	11.04^B^	11.46^B^	11.84^A^	12.10^A^
	16:00	35.17^D^	34.76^C^	36.28^aC^	35.66^bBC^	36.71^B^	36.68^AB^	37.72^A^	37.63^A^	38.09^A^	37.87^A^
	18:00	0.58	0.74	0.50	0.48	0.53^a^	0.47^b^	0.47	0.46	0.47	0.47
	**19:00**	**0.49**^**A**^	**0.50**	**0.52**^**A**^	**0.49**	**0.44**^**AB**^	**0.48**	**0.34**^**B**^	**0.41**	**0.46**^**AB**^	**0.48**
Cyclopropane	**17:0 cyclo ɷ7c**	**15.52**^**aB**^	**14.43**^**bB**^	**16.64**^**AB**^	**17.18**^**A**^	**16.87**^**A**^	**16.86**^**A**^	**17.40**^**A**^	**16.89**^**A**^	**16.38**^**AB**^	**16.22**^**A**^
	**19:0 cyclo ɷ8c**	**11.21**^**aC**^	**9.42**^**bB**^	**15.18**^**B**^	**15.45**^**A**^	**16.74**^**A**^	**15.88**^**A**^	**16.87**^**A**^	**16.34**^**A**^	**14.47**^**B**^	**14.54**^**A**^
Monounsaturated	**16:1 ɷ6c/16:1 ɷ7c**	**2.87**^**bA**^	**3.56**^**aA**^	**0.80**^**B**^	**0.82**^**B**^	**0.75**^**B**^	**0.70**^**B**^	**0.53**^**B**^	**0.57**^**B**^	**0.77**^**B**^	**0.70**^**B**^
	**17:1 ɷ7c**	**0.00**^**C**^	**0.00**^**B**^	**0.31**^**aA**^	**0.26**^**bA**^	**0.24**^**B**^	**0.30**^**A**^	**0.23**^**B**^	**0.27**^**A**^	**0.28**^**AB**^	**0.30**^**A**^
	**18:1 ɷ6c/18:1 ɷ7c**	**12.28**^**bA**^	**14.93**^**aA**^	**3.10**^**B**^	**3.31**^**B**^	**2.21**^**C**^	**2.30**^**C**^	**1.39**^**D**^	**1.47**^**D**^	**1.27**^**D**^	**1.23**^**D**^
	**18:1 ɷ7c 11-methyl**	**0.76**^**AB**^	**0.71**	**0.93**^**A**^	**0.83**	**0.76**^**AB**^	**0.84**	**0.57**^**B**^	**0.69**	**0.83**^**A**^	**0.86**
Polyunsaturated	20:2 ɷ6,9c	0.58	0.50	0.84	0.71	0.58	0.69	0.75	0.57	0.69	0.67
Hydroxy	**18:1 2OH**	**0.00**^**C**^	**0.00**^**B**^	**0.35**^**A**^	**0.31**^**A**^	**0.24**^**AB**^	**0.27**^**A**^	**0.20**^**B**^	**0.24**^**A**^	**0.29**^**AB**^	**0.30**^**A**^
Unresolved	14:0 3OH/16:1 iso I	8.05^BC^	7.97^BC^	9.51^A^	9.67^A^	8.74^AB^	8.68^B^	7.41^bC^	7.70^aC^	8.58^B^	8.66^BC^
	**18:0 anteiso/18:2 ɷ6,9c**	**1.01**^**A**^	**0.91**	**0.89**^**AB**^	**0.78**	**0.70**^**B**^	**0.77**	**0.60**^**B**^	**0.71**	**0.86**^**AB**^	**0.85**
	**19:1 ɷ6c/19:1 ɷ7c/19:0 cyclo**	**0.00**^**C**^	**0.00**^**B**^	**0.26**^**A**^	**0.22**^**A**^	**0.21**^**AB**^	**0.23**^**A**^	**0.18**^**B**^	**0.21**^**A**^	**0.21**^**AB**^	**0.24**^**A**^

The comparisons can be drawn among treatments or throughout time. Mean followed by different superscript lowercase letters in the same line (among treatments at the same time) and followed by different superscript uppercase letters in the columns (throughout time for each treatment, separately) differs at 5% probability (*p*-value < 0.05) by Tukey’s test. Where a letter is not shown, no statistical difference among samples was observed;

Main results discussed in the text are shown in bold.

The concentration of the fatty acids 19:00 and 18:1 ɷ7c 11-methyl, as well as, an unresolved mixture of the 18:0 anteiso/18:2 ɷ6,9c did not change throughout time of cultivation in the presence of C12-HSL, but changed in the control (Table 1). Additionally, after 6 h of cultivation, there was no difference in the concentration of the fatty acids 17:0 cyclo ɷ7c, 19:0 cyclo ɷ8c, 17:1 ɷ7c and 18:1 2OH, as well as, an unresolved mixture of the 19:1 ɷ6c/19:1 ɷ7c/19:0 cyclo in the treatment with C12-HSL (Table 1). Interestingly, the maintenance of the cyclopropane fatty acids 17:0 cyclo ɷ7c and 19:0 cyclo ɷ8c suggests that the cells are prepared for a possible stress condition. This hypothesis is strengthened by the fact that the cyclopropane fatty acids 17:0 cyclo and 19:0 cyclo are formed by transmethylation of *cis* monounsaturated fatty acids 16:1 ɷ7c and 18:1 ɷ7c, respectively, when the cell enters into stationary phase [65]. The level of cyclopropane fatty acids increases during the stationary phase due to the increased expression of the synthase that produces this fatty acid and is mediated by sigma S factor (σ^S^) [66, 67]. According to several studies, this modification may help reduce the impact of environmental stresses such as starvation, oxidation, acid and heavy metal addition, organic compound toxicity, increased temperature and pressure because it increases the stability and fluidity of the membrane, and at the same time reduces its permeability against toxic compounds [66–73]. An example of the effect of fatty acid modifications on bacterial cell membrane related to a stress condition was presented by Guckert et al. [68] who observed an increased proportion of cyclopropane fatty acids during nutrient deprivation in *Vibrio cholerae*.

**Fig 1:**
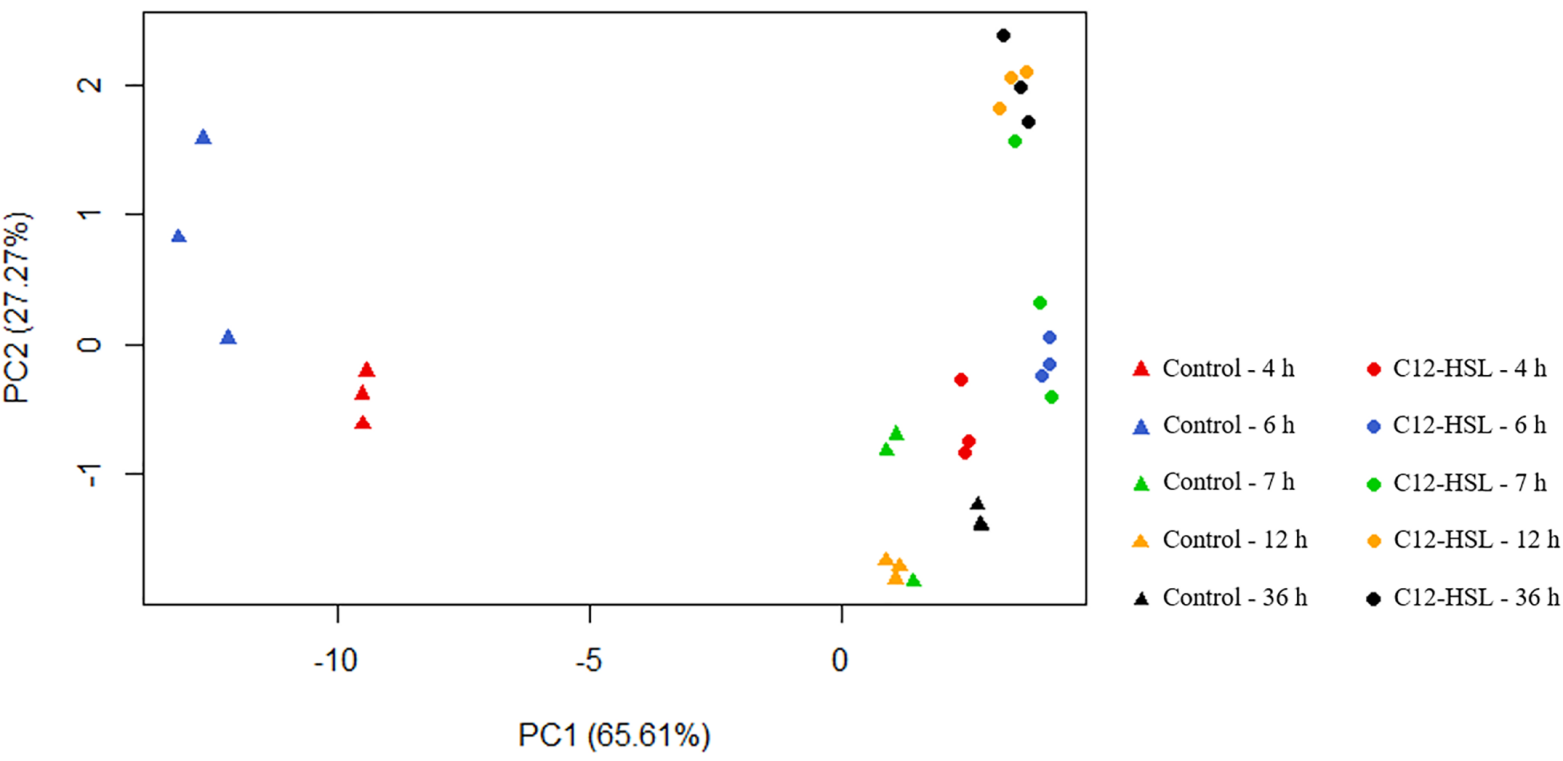
PCA analysis of fatty acids from *Salmonella* Enteritidis PT4 578 anaerobically cultivated in TSB at 37 °C in the presence or absence of C12-HSL. PCA of the percentages of each triplicate of fatty acid profile of *Salmonella* in the absence (control) and presence of C12-HSL.

The PCA was used in order to evaluate the variations among triplicates and to the understanding of the global difference of levels and types of fatty acids among samples stimulated by C12-HSL (Fig 1). The distance of the dots in the PCA figure (Fig 1) is proportional to the difference between the fatty acid composition of the experimental groups. Based on this information, there is a high dispersion between the global profile of fatty acids identified in the absence and presence of C12-HSL, which is represented by the distance between control and treatment at the same time. However, the graphic shows that the distance between control and treatment is higher at the 4 h and 6 h time points, with the dispersion tending to reduce over time as both groups tend to cluster more closely. On the other hand, within the treatment with C12-HSL, the identified fatty acids tended to be less dispersed throughout growth. Thus, the fatty acid profile of the cells in the logarithmic (4 h) and stationary (36 h) phases of growth were more similar to each other in the presence of AI-1 than in its absence.

The data also indicate that the fatty acid composition of *Salmonella* cells growing with C12-HSL for 4 h is similar to cells cultured in the absence of this autoinducer for 7 h (Fig 1). At this time of cultivation (7 h), the culture is already in the stationary phase of growth and the maintenance of the cyclopropane fatty acids suggest that the cells are prepared to support stressful conditions, as previously discussed. These results suggest that AHL signaling in *Salmonella* induces a profile of fatty acids which is typical to the stationary phase of growth, even in logarithmic phase of growth when the cell density was relatively low. According to Schuster et al. [74], this early preparation of the cells has a high fitness cost, but leads to more resistance should a stress condition arrive.

### 3.2. HSL alters the protein profile of *Salmonella* throughout time

The validation of proteins identifications showed percentages of FDR less than or equal to 0.8% for proteins (FDR ≤ 0.8%) and less than or equal to 0.14% for peptides (FDR ≤ 0.14%) (Table 2). These FDR values were lower than those accepted by Liu et al. [58] and Tran et al. [59], which admitted an FDR lower than 1% (FDR < 1.0%) for proteins and peptides of *Salmonella* Typhimurium.

**Table 2:** False discovery rates (FDR) for identification of proteins and peptides using decoy method by Scaffold software.

Time (h)	FDR (%)	
	Proteins	Peptides
4	0.7	0.14
6	0.7	0.06
7	0.0	0.00
12	0.7	0.14
36	0.8	0.05

FDR = False discovery rate.

**Fig 2:**
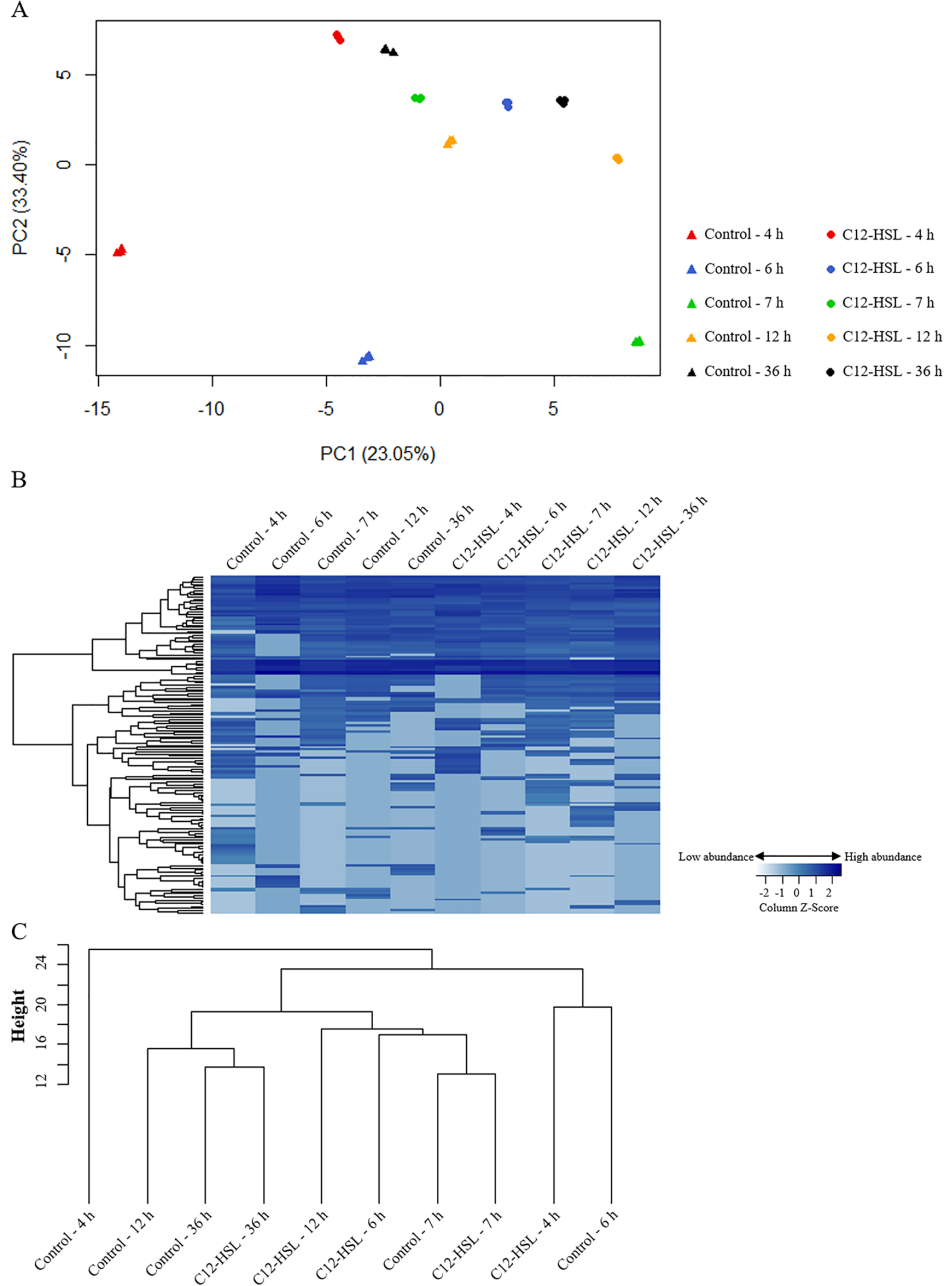
PCA, heatmap and dendrogram analyses of proteins from *Salmonella* Enteritidis PT4 578 anaerobically cultivated in TSB at 37 °C in the presence or absence of C12-HSL. **(A)** PCA of the logarithm of normalized TIC values of each triplicate of protein profile in the absence (control) and presence of C12-HSL. **(B)** Heatmap of the logarithm of normalized mean of TIC values of the triplicates for each identified protein. Each row corresponds to a unique protein, and each column the mean of the triplicate values. **(C)** Dendrogram of the logarithm normalized mean of TIC values of the triplicates for each identified protein. The height of the arms is proportional to the difference in the abundance profile of the proteins.

The protein composition at different time points is shown on Fig 2 and S2 Table. The PCA of the proteomic data showed that the variation among replicates was much lower than the variation among times (Fig 2A). There was a similar dispersion of the protein profile such as that observed with the fatty acid analysis, with a higher dispersion of protein and fatty acid profiles in the absence of C12-HSL (Fig 2A). The identified proteins in cells treated with C12-HSL tended to be less dispersed throughout growth than in the absence of the quorum sensing molecule (Fig 2A). Cluster analysis by agglomerative hierarchical methods of the proteins at the different time points was prepared generating a heatmap and a dendrogram that separated the proteins and the samples into two major clades, respectively (Fig 2B and 2C, respectively). Based on the color intensity variability in each column of the heatmap, the protein profile of *Salmonella* in the absence of C12-HSL at the 4 h time point presented more variations and, this was confirmed by the dendrogram (Fig 2C) that discriminates this sample from the others as a single branch in the tree.

Interestingly, the PCA, the heatmap and the dendrogram analyses together showed that the addition of C12-HSL at the beginning of *Salmonella* cultivation resulted in smaller protein profile variations throughout the time of cultivation when compared with the control. Thus, the protein profile of the cells in the logarithmic (4 h) and stationary (36 h) phases of growth were more similar to each other in the presence of AI-1 than in its absence as well as the fatty acid profile previously shown. Based on these similarities, these results suggest that AI-1 can anticipate a stationary phase response. Moreover, these results corroborate our previous observation, in which the differentially abundant proteins and organic acids of *Salmonella* cultivated for 7 h in the presence of C12-HSL correlated with entry into the stationary phase of growth, mainly in relation to nitrogen and amino acid starvation as well as acid stress [35]. Schuster et al. [36] showed that genes with expression influenced by the growth phase in *P*. *aeruginosa* were repressed by quorum sensing during the late logarithmic and stationary phases. Goo et al. [38] showed that quorum sensing anticipates and influences the survival to the stress of the stationary phase of *B*. *glumae*, *B*. *pseudomallei* and *B*. *thailandensis*. The survival of these bacteria requires the activation of cellular enzymes through the quorum sensing mechanism for production of excreted oxalate, which serves to counteract ammonia-mediated alkaline toxicity during stationary phase [38]. In addition, the genes involved in transcription and translation of *B*. *pseudomallei* in stationary phase were co-regulated by RpoS protein (RNA polymerase sigma factor) and quorum sensing [75].

The statistical analyses of the identified proteins are shown in Table 3 and S3 Table. The results showed that at 4 and 6 h of incubation in presence of C12-HSL, a higher percentage of differentially abundant proteins was observed in comparison with other times of cultivation (Table 4). In addition, more proteins had their abundance decreased at 4 h in the presence of AHL (50.0%), while an opposite trend was observed at 6 h (54.8%). The greatest number of differentially abundant proteins at the initial times can be due to the addition of AI-1 at the beginning of the cultivation. Growth and cell size are altered by intrinsic and extrinsic factors during the adaptation phase to an environmental condition and, consequently, alter the cellular components resulting in a greater fluctuation in protein abundance at initial times of cultivation [76, 77]. In AHL-producing bacteria, such as *B*. *thailandensis* and *P*. *aeruginosa*, many genes were regulated at the end of the logarithmic and beginning of the stationary phase of growth due to the accumulation of signaling molecules in the medium [36, 37, 78]. Schuster et al. [36] showed that genes involved in carbohydrate utilization or nutrient transport were the most repressed by quorum sensing during the late logarithmic and stationary phases of *P*. *aeruginosa*.

**Table 3:** Statistical analysis of the abundance of proteins identified from *Salmonella* Enteritidis PT4 578 anaerobically cultivated in TSB at 37 °C in the presence or absence of C12-HSL.

Protein	Protein name	Gene	Process	Time (h)									
				4		6		7		12		36	
				Log_2_ FC	-Log_10_ *p*	Log_2_ FC	-Log_10_ *p*	Log_2_ FC	-Log_10_ *p*	Log_2_ FC	-Log_10_ *p*	Log_2_ FC	-Log_10_ *p*
ATP synthase subunit delta	Q7CPE5	*atpH*	Biosynthetic	-11.566	1.060	8.687	3.470	**1.157**	**2.760**	ND	ND	ND	ND
Acetyl-CoA carboxylase, BCCP subunit	Q7CPM1	*accB*	Biosynthetic	2.049	0.841	ND	ND	-10.547	1.542	ND	ND	-1.505	1.241
Acyl carrier protein	P0A6B1	*acpP*	Biosynthetic	1.930	1.139	-0.409	0.737	-0.439	0.714	-0.277	1.882	-0.452	1.297
ATP synthase subunit beta	Q7CPE2	*atpD*	Biosynthetic	ND	ND	ND	ND	-7.974	1.357	ND	ND	ND	ND
Cell division protein FtsZ	Q8ZRU0	*ftsZ*	Cell Division	ND	ND	ND	ND	-7.378	1.542	8.561	1.296	ND	ND
Cell division protein ZapB	Q8ZKP1	*zapB*	Cell Division	-9.554	2.451	10.650	2.037	-0.305	0.317	-0.300	0.554	0.912	1.133
Protein phosphatase CheZ	P07800	*cheZ*	Chemotaxis	-8.243	4.181	2.733	0.940	3.359	0.506	0.029	0.023	9.365	1.026
Aspartate ammonia-lyase	Q7CPA1	*aspA*	Metabolic process	ND	ND	ND	ND	6.377	1.508	ND	ND	ND	ND
Arginine decarboxylase	Q8ZKE3	*adi*	Metabolic process	ND	ND	ND	ND	ND	ND	ND	ND	-8.168	1.714
Shikimate kinase 1	P63601	*aroK*	Metabolic process	-10.245	2.324	7.418	1.665	0.677	0.288	**1.517**	**1.882**	ND	ND
2-iminobutanoate/2-iminopropanoate deaminase	Q7CP78	*ridA*	Metabolic process	2.789	1.273	**-3.083**	**3.151**	-0.235	0.865	-2.437	0.866	-0.781	1.249
Autonomous glycyl radical cofactor	Q7CQ05	*grcA*	Metabolic process	**0.816**	**1.306**	**0.940**	**2.063**	0.511	1.542	0.072	0.162	-0.513	1.714
Pyruvate formate lyase I, induced anaerobically	Q7CQU1	*pflB*	Metabolic process	ND	ND	11.573	0.825	-0.159	1.542	ND	ND	ND	ND
Lactoylglutathione lyase	P0A1Q2	*gloA*	Metabolic process	-6.833	0.914	ND	ND	ND	ND	ND	ND	ND	ND
Enolase	P64076	*eno*	Metabolic process	**-1.074**	**1.640**	-0.551	0.328	-0.417	0.506	1.104	0.668	0.180	0.140
Glyceraldehyde-3-phosphate dehydrogenase	P0A1P0	*gapA*	Metabolic process	ND	ND	ND	ND	ND	ND	9.405	1.107	ND	ND
Phosphoglycerate kinase	P65702	*pgk*	Metabolic process	-0.310	0.812	0.108	0.113	**0.746**	**1.988**	-0.487	1.719	0.562	1.987
Triosephosphate isomerase	Q8ZKP7	*tpiA*	Metabolic process	-11.938	2.806	-11.340	2.011	-0.216	0.169	8.235	0.996	ND	ND
Adenylate kinase	P0A1V4	*adk*	Metabolic process	0.221	0.329	**1.820**	**1.810**	0.091	0.215	-0.451	1.037	-1.271	1.260
Deoxyribose-phosphate aldolase	Q8ZJV8	*deoC*	Metabolic process	-7.031	0.839	ND	ND	-0.630	0.925	ND	ND	7.796	0.767
Pyrimidine/purine nucleoside phosphorylase	Q8ZRE7	*ppnP*	Metabolic process	ND	ND	ND	ND	ND	ND	8.356	1.530	ND	ND
Acetate kinase	P63411	*ackA*	Metabolic process	-8.530	2.435	9.037	2.391	0.800	0.511	8.469	1.882	ND	ND
Ribose-5-phosphate isomerase A	P66692	*rpiA*	Metabolic process	-8.886	3.352	ND	ND	0.426	0.481	ND	ND	-0.871	1.020
6,7-dimethyl-8-ribityllumazine synthase	P66038	*ribH*	Metabolic process	-10.296	2.100	8.466	2.208	-0.989	0.842	-0.515	1.415	9.901	1.243
Flagellar hook protein FlgE	P0A1J1	*flgE*	Motility	ND	ND	ND	ND	-8.339	1.048	ND	ND	-6.615	1.054
Flagellar hook-associated protein 3	P16326	*flgL*	Motility	-6.794	1.152	ND	ND	ND	ND	ND	ND	ND	ND
Flagella synthesis protein FlgN	P0A1J7	*flgN*	Motility	-9.157	1.970	ND	ND	-0.528	0.735	-0.138	0.125	8.395	4.679
Flagellar hook-associated protein 2	B5R7H2	*fliD*	Motility	ND	ND	ND	ND	7.292	2.329	ND	ND	ND	ND
Flagellin	B5R7H3	*fljB*	Motility	0.012	0.034	-0.297	1.298	0.179	1.542	-0.076	0.940	0.316	1.016
[2FE-2S] ferredoxin	Q7CQ13	*fdx*	Oxidation-reduction	-0.511	0.404	ND	ND	10.435	2.995	**1.162**	**1.696**	-8.019	1.577
Flavodoxin 1	Q8ZQX1	*fldA*	Oxidation-reduction	ND	ND	ND	ND	ND	ND	9.103	1.504	9.307	1.054
Glutaredoxin 1	P0A1P8	*grxA*	Oxidation-reduction	ND	ND	10.883	0.737	ND	ND	ND	ND	-0.839	0.547
Glutaredoxin 3	Q7CPH7	*grxC*	Oxidation-reduction	ND	ND	ND	ND	ND	ND	ND	ND	8.107	1.078
Hydrogenase-3, iron-sulfur subunit	Q7CPY1	*hycB*	Oxidation-reduction	-7.993	1.393	ND	ND	ND	ND	ND	ND	ND	ND
Protease involved in processing C-terminal end of HycE	Q8ZMJ3	*hycI*	Oxidation-reduction	ND	ND	ND	ND	0.469	0.605	-1.598	1.076	0.655	0.668
Glutaredoxin	Q7CQK9	*ydhD*	Oxidation-reduction	-1.224	0.976	9.653	1.513	-0.180	1.357	1.196	1.179	-0.311	1.054
Alkyl hydroperoxide reductase subunit C	P0A251	*ahpC*	Oxidation-reduction	-0.080	0.233	**0.592**	**1.393**	-0.625	1.154	0.305	0.488	-0.400	0.430
Thioredoxin dependent thiol peroxidase	Q7CQ23	*bcp*	Oxidation-reduction	ND	ND	ND	ND	ND	ND	ND	ND	8.506	1.054
Thiol:disulfide interchange protein DsbC	P55890	*dsbC*	Oxidation-reduction	ND	ND	-8.491	0.940	ND	ND	ND	ND	ND	ND
Oxygen-insensitive NAD(P)H nitroreductase	P15888	*nfsB*	Oxidation-reduction	8.325	4.181	9.274	1.111	**0.590**	**1.542**	9.311	1.719	**1.708**	**2.151**
Superoxide dismutase [Fe]	P0A2F4	*sodB*	Oxidation-reduction	ND	ND	ND	ND	**1.417**	**1.979**	-1.254	0.738	ND	ND
Superoxide dismutase [Cu-Zn] 1	P0CW86	*sodC1*	Oxidation-reduction	11.567	2.324	0.060	0.082	-0.093	0.098	0.866	1.107	0.151	0.747
Putative thiol-alkyl hydroperoxide reductase	Q7CR42	STM0402	Oxidation-reduction	**-0.931**	**3.817**	**1.287**	**1.810**	-0.538	1.542	-0.294	0.689	-0.746	0.767
Putative thiol-disulfide isomerase and thioredoxin	Q8ZP25	STM1790	Oxidation-reduction	-9.429	1.436	10.287	2.011	**0.629**	**2.192**	**1.026**	**1.719**	-0.532	1.000
Probable thiol peroxidase	Q8ZP65	*tpx*	Oxidation-reduction	-9.378	1.761	**1.394**	**1.606**	0.131	0.434	0.191	0.911	0.295	0.968
Thioredoxin 1	P0AA28	*trxA*	Oxidation-reduction	0.284	0.274	-0.518	0.812	**0.792**	**1.542**	**-1.247**	**1.525**	-0.567	1.836
Arsenate reductase	Q8ZN68	*yfgD*	Oxidation-reduction	-9.881	1.761	9.480	1.565	-0.553	1.154	-0.854	1.152	-0.491	0.668
Fimbrial protein	P12061	*sefA*	Pathogenesis	**1.178**	**3.123**	-0.109	0.677	-0.236	3.658	-0.321	1.359	-0.228	0.747
Non-specific acid phosphatase	P26976	*phoN*	Pathogenesis	ND	ND	-9.715	2.323	ND	ND	ND	ND	ND	ND
Major outer membrane lipoprotein 1	Q7CQN4	*lpp1*	Pathogenesis	ND	ND	-8.366	1.046	ND	ND	ND	ND	ND	ND
Ecotin	Q8ZNH4	*eco*	Pathogenesis	**1.515**	**1.561**	10.403	1.629	0.246	3.740	-0.399	0.571	**1.421**	**3.339**
Peptidyl-prolyl cis-trans isomerase	Q8ZLL6	*fkpA*	Protein folding	**1.695**	**2.381**	0.213	1.810	-0.265	1.501	**0.945**	**1.415**	**-0.773**	**1.304**
Peptidyl-prolyl cis-trans isomerase	Q8XFG8	*ppiB*	Protein folding	-8.726	2.358	7.501	1.665	ND	ND	ND	ND	ND	ND
Peptidyl-prolyl cis-trans isomerase	Q8ZLL4	*slyD*	Protein folding	0.401	1.152	1.340	1.072	**0.691**	**1.542**	0.560	0.883	-0.362	0.404
Trigger fator	P66932	*tig*	Protein folding	-1.402	1.238	0.665	0.451	0.708	1.105	-0.426	1.485	**-0.730**	**1.971**
Chaperone protein DnaK	Q56073	*dnaK*	Protein folding	**-2.246**	**1.390**	0.407	0.928	-0.040	0.142	0.166	0.289	-0.627	0.809
60 kDa chaperonin	P0A1D3	*groL*	Protein folding	0.758	0.661	-0.350	0.139	**2.690**	**4.349**	-1.241	0.765	-0.108	0.067
10 kDa chaperonin	P0A1D5	*groS*	Protein folding	0.969	1.188	0.433	0.865	0.037	0.178	0.076	0.585	0.182	1.840
Protein GrpE	Q7CPZ4	*grpE*	Protein folding	-9.471	1.122	7.826	0.799	8.528	0.842	ND	ND	**-1.209**	**1.972**
Small heat shock protein IbpA	Q7CPF1	*ibpA*	Protein folding	ND	ND	ND	ND	ND	ND	ND	ND	-7.675	1.054
Chaperone protein Skp	P0A1Z2	*skp*	Protein folding	ND	ND	ND	ND	-10.241	1.299	9.656	0.990	ND	ND
Chaperone SurA	Q7CR87	*surA*	Protein folding	-7.111	2.324	ND	ND	ND	ND	ND	ND	ND	ND
Iron-sulfur cluster insertion protein ErpA	Q7CR66	*erpA*	Protein maturation	ND	ND	-7.504	1.598	ND	ND	ND	ND	ND	ND
Fe/S biogenesis protein NfuA	Q8ZLI7	*nfuA*	Protein maturation	**2.317**	**1.761**	0.908	0.593	-0.996	1.048	-1.033	1.106	-0.700	0.767
Iron-sulfur cluster assembly scaffold protein IscU	Q7CQ11	*nifU*	Protein maturation	9.913	1.176	**-1.624**	**1.512**	-8.446	1.151	9.074	1.271	ND	ND
S-ribosylhomocysteine lyase	Q9L4T0	*luxS*	Quorum sensing	-0.240	0.614	11.588	1.665	0.421	0.914	0.761	1.076	0.039	0.097
Single-stranded DNA-binding protein 1	P0A2F6	*ssb*	Response to stress	-7.772	1.761	ND	ND	8.250	1.368	ND	ND	ND	ND
UPF0234 protein YajQ	Q8ZRC9	*yajQ*	Response to stress	-7.420	2.808	8.948	0.625	ND	ND	ND	ND	ND	ND
Universal stress protein G	P67093	*uspG*	Response to stress	ND	ND	9.407	2.079	0.013	0.039	1.175	1.107	0.198	1.026
Putative outer membrane protein	Q7CPS4	*ygiW*	Response to stress	ND	ND	6.498	1.320	-7.298	2.011	-6.702	1.296	ND	ND
Putative molecular chaperone (Small heat shock protein)	Q8ZPY6	STM1251	Response to stress	ND	ND	ND	ND	ND	ND	9.601	1.882	0.157	0.078
RNA polymerase-binding transcription factor DksA	P0A1G5	*dksA*	Transcription	ND	ND	ND	ND	7.810	2.307	8.954	0.727	-7.468	2.038
Cold shock-like protein CspC	P0A9Y9	*cspC*	Transcription	11.820	1.185	0.813	0.987	**2.846**	**2.329**	-1.023	0.571	-0.194	0.767
RNA chaperone, negative regulator of cspA transcription	Q7CQZ5	*cspE*	Transcription	ND	ND	-8.997	1.858	ND	ND	ND	ND	-8.747	1.840
Transcriptional repressor of emrAB operon	Q7CPY9	*emrR*	Transcription	-7.967	1.060	7.639	2.079	7.947	1.745	7.095	1.485	ND	ND
Transcriptional repressor of iron-responsive genes (Fur family) (Ferric uptake regulator)	Q7CQY3	*fur*	Transcription	0.175	0.574	8.426	1.998	-0.134	0.248	0.544	1.076	0.459	0.253
Transcription elongation factor GreA	P64281	*greA*	Transcription	**-1.164**	**1.352**	10.895	1.810	-0.544	0.776	-0.501	1.754	0.006	0.011
DNA-binding protein H-NS	P0A1S2	*hns*	Transcription	**-3.346**	**2.381**	-1.089	0.898	0.174	0.248	0.042	0.022	-0.278	0.809
DNA-binding protein HU-alpha	P0A1R6	*hupA*	Transcription	**1.714**	**2.363**	0.140	0.593	**0.646**	**1.729**	0.940	0.990	**-0.633**	**1.826**
Virulence transcriptional regulatory protein PhoP	P0DM78	*phoP*	Transcription	-10.751	1.390	8.923	1.046	**0.758**	**2.854**	0.030	0.019	**0.955**	**5.924**
Regulator of nucleoside diphosphate kinase	Q7CQZ7	*rnk*	Transcription	-7.467	2.747	ND	ND	-0.012	0.019	-6.467	1.037	ND	ND
DNA-directed RNA polymerase subunit alpha	P0A7Z7	*rpoA*	Transcription	-0.255	2.435	**2.402**	**1.998**	-0.135	0.851	-1.706	0.561	-1.425	1.297
DNA-directed RNA polymerase subunit omega	P0A803	*rpoZ*	Transcription	**-1.482**	**1.796**	8.487	1.998	**-1.524**	**2.995**	0.738	0.554	**-0.963**	**2.151**
DNA-binding protein	B5RBI8	SG2019	Transcription	-7.989	2.381	ND	ND	6.415	2.307	0.535	0.328	ND	ND
Transcriptional regulator SlyA	P40676	*slyA*	Transcription	ND	ND	ND	ND	-0.341	0.142	-8.648	1.327	-2.417	1.026
DNA-binding protein StpA	P0A1S4	*stpA*	Transcription	0.152	0.125	ND	ND	ND	ND	8.362	3.372	ND	ND
Nucleoid-associated protein YbaB	P0A8B8	*ybaB*	Transcription	0.454	0.416	0.312	0.255	-0.286	0.349	-9.610	1.037	-0.817	1.249
Transcription modulator YdgT	Q7CQK5	*ydgT*	Transcription	-9.106	2.324	ND	ND	ND	ND	ND	ND	ND	ND
Inorganic pyrophosphatase	P65748	*ppa*	Transcription	-10.551	1.376	-2.442	0.737	0.540	0.598	0.730	0.480	**-0.911**	**2.335**
Modulator of Rho-dependent transcription termination	Q8ZRN4	*rof*	Transcription	ND	ND	ND	ND	0.720	1.072	-0.815	0.616	-0.751	0.981
Regulator of ribonuclease activity A	P67651	*rraA*	Transcription	ND	ND	ND	ND	7.518	2.329	ND	ND	ND	ND
Stringent starvation protein B	Q7CPN4	*sspB*	Transcription	ND	ND	ND	ND	7.192	2.063	ND	ND	ND	ND
Peptide deformylase	Q8ZLM7	*def*	Translation	-8.092	2.806	ND	ND	-8.530	1.503	-8.086	1.719	ND	ND
Ribosome recycling fator	P66738	*rrf or frr*	Translation	**-2.277**	**3.208**	**0.989**	**1.558**	0.444	0.506	**1.071**	**1.330**	0.602	1.026
Stationary-phase-induced ribosome-associated protein	Q7CQJ0	*sra*	Translation	ND	ND	-9.400	2.414	-9.252	1.151	0.305	0.238	**-2.258**	**1.840**
Ribosome associated fator	Q7CQ00	*yfiA*	Translation	ND	ND	-7.943	2.079	ND	ND	-8.664	2.295	-9.657	1.387
Translation initiation factor IF-1	P69226	*infA*	Translation	ND	ND	ND	ND	8.689	0.617	7.127	1.719	ND	ND
Translation initiation factor IF-3	P33321	*infC*	Translation	-10.818	1.790	8.352	1.512	ND	ND	-7.574	1.754	-7.938	2.417
50S ribosomal protein L1	P0A2A3	*rplA*	Translation	-0.903	1.148	-8.794	2.134	9.181	1.039	ND	ND	ND	ND
50S ribosomal protein L3	P60446	*rplC*	Translation	9.334	2.324	ND	ND	ND	ND	ND	ND	ND	ND
50S ribosomal protein L6	P66313	*rplF*	Translation	**0.593**	**1.957**	-0.186	0.328	ND	ND	7.572	0.841	-9.383	1.241
50S ribosomal protein L9	Q8ZK80	*rplI*	Translation	0.306	2.454	-0.381	1.665	0.147	0.183	**-0.995**	**1.359**	-0.361	1.026
50S ribosomal protein L10	P0A297	*rplJ*	Translation	-0.425	0.870	9.051	2.011	0.557	0.637	**1.216**	**1.396**	9.329	1.220
50S ribosomal protein L11	P0A7K0	*rplK*	Translation	0.564	1.152	-9.100	1.288	9.783	0.846	ND	ND	-8.354	1.971
50S ribosomal protein L7/L12	P0A299	*rplL*	Translation	1.043	1.148	**0.832**	**1.665**	0.408	0.615	-1.167	0.990	0.692	0.668
50S ribosomal protein L15	P66073	*rplO*	Translation	-10.938	4.151	ND	ND	ND	ND	ND	ND	ND	ND
50S ribosomal protein L17	Q7CPL7	*rplQ*	Translation	**-0.782**	**1.990**	**3.526**	**4.757**	0.467	0.466	9.427	0.677	-0.123	0.055
50S ribosomal protein L18	Q7CPL6	*rplR*	Translation	**1.375**	**2.324**	ND	ND	-0.275	0.235	9.705	1.182	ND	ND
50S ribosomal protein L19	P0A2A1	*rplS*	Translation	-0.533	0.420	ND	ND	ND	ND	10.169	0.909	ND	ND
50S ribosomal protein L24	P60626	*rplX*	Translation	-0.441	0.873	11.243	3.470	1.216	0.773	-0.730	1.107	0.442	0.305
50S ribosomal protein L25	Q7CQ71	*rplY*	Translation	-0.776	1.230	7.944	1.598	**0.984**	**1.542**	0.793	0.382	-0.605	0.707
50S ribosomal protein L28	P0A2A5	*rpmB*	Translation	**-1.746**	**2.358**	9.704	1.095	0.537	0.297	8.210	1.025	ND	ND
50S ribosomal protein L29	P66170	*rpmC*	Translation	**-1.525**	**1.640**	8.702	1.677	1.191	0.481	**-0.900**	**2.043**	0.539	0.280
50S ribosomal protein L30	P0A2A7	*rpmD*	Translation	ND	ND	ND	ND	ND	ND	ND	ND	-9.619	2.532
50S ribosomal protein L31	P66191	*rpmE*	Translation	ND	ND	ND	ND	ND	ND	10.159	1.074	ND	ND
50S ribosomal protein L33	P0A7P2	*rpmG*	Translation	ND	ND	8.313	2.037	ND	ND	7.849	1.076	ND	ND
50S ribosomal protein L34	P0A7P8	*rpmH*	Translation	-7.903	2.324	ND	ND	ND	ND	9.446	1.053	ND	ND
30S ribosomal protein S1	Q7CQT9	*rpsA*	Translation	**-3.033**	**4.181**	ND	ND	**1.901**	**1.525**	-1.215	0.505	ND	ND
30S ribosomal protein S2	P66541	*rpsB*	Translation	-7.096	1.761	ND	ND	ND	ND	ND	ND	ND	ND
30S ribosomal protein S4	O54297	*rpsD*	Translation	-0.643	0.937	ND	ND	ND	ND	8.864	0.990	ND	ND
30S ribosomal protein S5	P0A7W4	*rpsE*	Translation	-1.050	0.941	-8.356	1.776	ND	ND	ND	ND	ND	ND
30S ribosomal protein S6	P66593	*rpsF*	Translation	-9.552	1.680	6.762	1.521	0.510	0.344	ND	ND	ND	ND
30S ribosomal protein S7	P0A2B3	*rpsG*	Translation	-0.345	1.761	ND	ND	-0.539	0.666	ND	ND	ND	ND
30S ribosomal protein S8	P0A7X0	*rpsH*	Translation	**-1.382**	**2.297**	**2.600**	**2.902**	**0.735**	**1.542**	0.112	0.285	**-2.163**	**2.532**
30S ribosomal protein S10	P67904	*rpsJ*	Translation	0.303	0.245	-0.520	0.495	0.125	0.142	-1.227	0.585	ND	ND
30S ribosomal protein S11	O54296	*rpsK*	Translation	1.423	1.139	ND	ND	ND	ND	ND	ND	ND	ND
30S ribosomal protein S13	Q8ZLM1	*rpsM*	Translation	0.283	0.242	ND	ND	ND	ND	ND	ND	ND	ND
30S ribosomal protein S14	P66409	*rpsN*	Translation	-0.353	0.568	7.881	1.810	-0.451	2.036	2.427	0.990	ND	ND
30S ribosomal protein S18	Q8ZK81	*rpsR*	Translation	-12.161	2.208	ND	ND	ND	ND	ND	ND	ND	ND
30S ribosomal protein S19	P66491	*rpsS*	Translation	0.149	0.404	ND	ND	ND	ND	ND	ND	ND	ND
30S ribosomal protein S20	P0A2B1	*rpsT*	Translation	**1.295**	**4.181**	**1.915**	**1.348**	0.834	1.072	1.105	0.776	-1.148	1.054
Elongation factor P	P64036	*efp*	Translation	-9.275	1.432	ND	ND	ND	ND	ND	ND	ND	ND
Elongation factor Ts	P64052	*tsf*	Translation	-0.212	0.451	**1.346**	**1.596**	0.609	1.154	**-0.872**	**1.754**	**-2.134**	**2.151**
Elongation factor Tu	P0A1H5	*tufA*	Translation	**-3.968**	**1.957**	**2.390**	**2.077**	-0.543	0.598	-0.425	0.147	-0.140	0.096
Histidine-binding periplasmic protein	P02910	*hisJ*	Transport	-9.017	1.957	-1.073	0.737	-0.934	0.522	9.351	1.331	0.646	0.920
PTS system glucose-specific EIIA component	P0A283	*crr*	Transport	-0.304	0.841	**-2.267**	**3.151**	**-1.073**	**1.822**	-0.399	1.485	0.063	0.257
Multiphosphoryl transfer protein	P17127	*fruB*	Transport	ND	ND	-7.368	1.542	ND	ND	ND	ND	ND	ND
Phosphocarrier protein HPr	P0AA07	*ptsH*	Transport	8.437	1.230	7.848	3.151	0.504	0.481	1.948	0.711	-9.520	0.737
Glutamine high-affinity transporter	Q7CQW0	*glnH*	Transport	ND	ND	ND	ND	ND	ND	-7.105	1.102	ND	ND
Ferritin	Q8ZNU4	*ftn*	Transport	-10.668	2.044	9.884	2.902	1.148	0.945	ND	ND	10.071	1.054
Protein-export protein SecB	Q7CPH8	*secB*	Transport	-7.460	1.409	10.905	1.252	ND	ND	ND	ND	ND	ND
Outer membrane protein A	P02936	*ompA*	Transport	**1.039**	**2.044**	0.063	0.113	-0.312	0.482	0.140	0.207	**0.693**	**1.987**
Outer membrane channel	Q8ZLZ4	*tolC*	Transport	ND	ND	ND	ND	10.139	0.594	ND	ND	-0.744	0.767
BssS protein Family	A0A1V9AFN8	ABA47_0691	Unclassified	-8.706	2.381	ND	ND	0.807	0.663	0.364	0.253	ND	ND
Uncharacterized protein	A0A1F2JWR0	HMPREF 3126_08675	Unclassified	ND	ND	ND	ND	ND	ND	-9.642	1.719	ND	ND
Uncharacterized protein	A0A1R2IBX3	R567_04560	Unclassified	ND	ND	ND	ND	8.671	0.842	ND	ND	-7.007	0.967
Uncharacterized protein	B5RBG9	SG1997	Unclassified	-7.908	1.098	-9.302	1.665	ND	ND	7.916	0.779	ND	ND
Putative periplasmic protein	Q8ZPY8	STM1249	Unclassified	ND	ND	ND	ND	1.332	1.094	7.565	1.882	9.917	1.431
UPF0253 protein YaeP	P67551	*yaeP*	Unclassified	ND	ND	ND	ND	8.765	1.985	ND	ND	ND	ND
Putative cytoplasmic protein	Q8ZQ41	*yccJ*	Unclassified	ND	ND	ND	ND	9.189	0.663	9.850	1.719	-9.249	2.067
Uncharacterized protein	Q7CQR0	*ycfF*	Unclassified	ND	ND	ND	ND	ND	ND	7.127	1.107	ND	ND
Putative cytoplasmic protein	Q7CQJ6	*ydfZ*	Unclassified	-1.164	1.244	**0.884**	**2.134**	0.262	2.329	-0.047	0.221	-0.048	0.063
Putative cytoplasmic protein	Q7CQB7	*yecF*	Unclassified	-9.069	0.706	ND	ND	ND	ND	ND	ND	6.772	1.342
UPF0265 protein YeeX	P67605	*yeeX*	Unclassified	-12.867	2.813	10.455	1.629	1.293	0.890	-0.540	0.696	8.884	1.714
Putative cytoplasmic protein	Q7CQ33	*yfcZ*	Unclassified	**1.467**	**1.409**	-0.175	0.344	0.415	1.314	**-0.656**	**1.562**	0.010	0.029
Putative cytoplasmic protein	Q8ZKH3	*yjbR*	Unclassified	ND	ND	ND	ND	ND	ND	7.963	1.687	ND	ND
Uncharacterized protein	I3W485	-	Unclassified	ND	ND	8.155	1.028	ND	ND	8.028	0.859	ND	ND

ND = not detected;

Log_2_ FC = logarithm in base two of fold changed (ratio of the normalized mean of TIC values of the treatment with C12-HSL by the control);

-Log_10_ *p* = negative logarithm of *p*-value;

Gray background and bold number = increased abundance of protein in C12-HSL and detected in both treatments (Log_2_ FC > 0.585 and -Log_10_ > 1.301);

Gray background = increased abundance of protein in C12-HSL and detected only in the treatment with C12-HSL (Log_2_ FC > 0.585);

Yellow background and bold number = decreased abundance of protein in C12-HSL and detected in both treatments (Log_2_ FC < -0.585 and -Log_10_ > 1.301);

Yellow background = decreased abundance of protein in C12-HSL and detected only in the control treatment (Log_2_ FC < -0.585).

**Table 4:** Number and percentage of differentially abundant proteins in comparison to the total proteins identified from *Salmonella* Enteritidis PT4 578 anaerobically cultivated in TSB at 37 °C in the presence C12-HSL.

Time (h)	Proteins identified						
	Abundance increased		Abundance decreased		Differentially abundant		Total
	Number	%	Number	%	Number	%	Number
4	17	15.5	55	50.0	72	65.5	110
6	51	54.8	16	17.2	67	72.0	93
7	31	28.7	11	10.2	42	38.9	108
12	36	34.0	14	13.2	50	47.2	106
36	16	17.8	23	25.6	39	43.4	90

**Fig 3:**
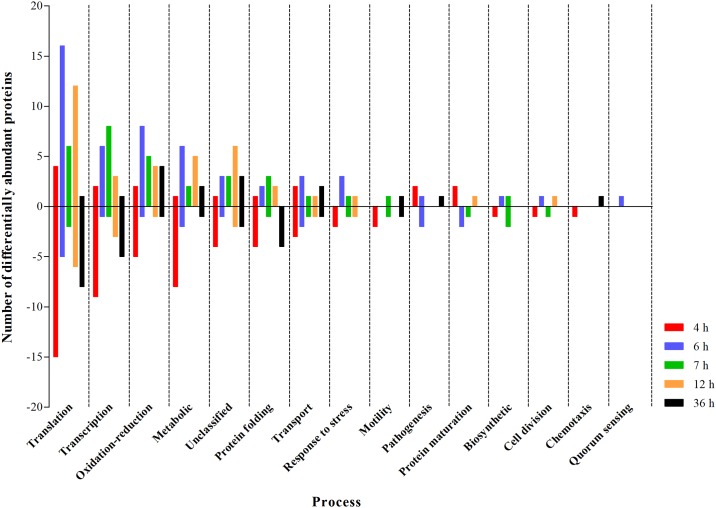
Number of differentially abundant proteins grouped according to the process on Gene Ontology (GO) annotations (European Bioinformatics Institute). The Y-axis represents the number of differentially abundant proteins: above zero the number of proteins in which the abundance increased in the presence of C12-HSL compared to the control and below zero represents the proteins in which the abundance decreased in the presence of C12-HSL.

The differentially abundant proteins were grouped in order to perform an analysis of enrichment of biological processes based on GO annotations, as shown in Fig 3. The proteins related to translation, transcription, oxidation-reduction, metabolic, protein folding, transport processes as well as unclassified proteins, had their abundance affected by C12-HSL in all the studied time points.

An important identified protein is LuxS (S-ribosylhomocysteine lyase), which produces the autoinducer-2 signaling molecule (Table 3 and Fig 3). In our experimental conditions, this protein was identified only in the presence of 50 nM of C12-HSL at 6 h of cultivation, that is, at the end of the logarithmic phase of growth of *Salmonella*. This result suggests that there could be a cross response between quorum sensing mechanisms mediated by AI-1 and AI-2 in *Salmonella* depending on the growth phase. Interactions among the different mechanisms of quorum sensing present in *P*. *aeruginosa* have been described leading to a hierarchical activation of these mechanisms [79–81] and also to the synthesis of inductive and inhibitory molecules [82]. The existence of multiple arrangements between the different mechanisms of quorum sensing might play an important role in processing of environmental cues and thus, dictating necessary and robust collective responses [83]. Additional studies should be performed in *Salmonella* to confirm this possible connection.

**Fig 4:**
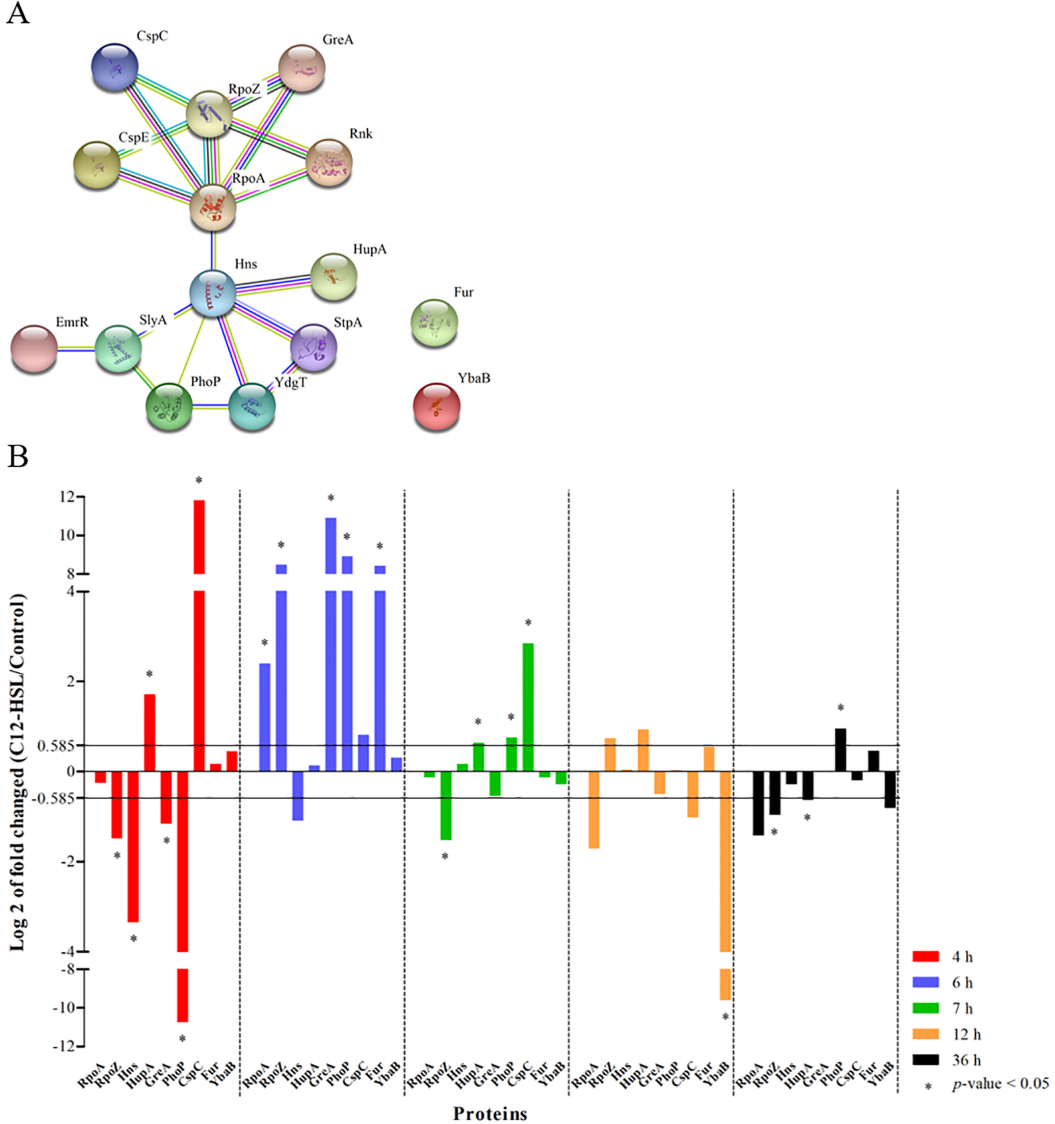
Proteins related to transcription process and “regulation of transcription, DNA-templated” function of *Salmonella* Enteritidis PT4 578, anaerobically cultivated in TSB at 37 °C in the presence or absence of C12-HSL. **(A)** The network of interactions among the proteins of the transcription process and “regulation of transcription, DNA-templated” function and **(B)** the logarithm in base two of fold changed of the proteins that were identified at all times and in at least one of the treatments.

Among the identified proteins, a greater number was involved in transcription process showed a variation of their abundance in the presence of C12-HSL (Fig 3). Since transcription is an essential step in gene expression and the transcriptional regulation determines the molecular machinery for developmental plasticity, homeostasis and adaptation [84], a network of interaction between the proteins related to the transcription process and “regulation of transcription, DNA-templated” was generated (Fig 4A). The PPI network showed an average local clustering coefficient of 0.636 and a *p*-value of <1.06e-10 for enrichment, indicating that the interactions showed at medium confidence or better and, the proteins have more interactions among themselves than what would have been expected for a random set of proteins of similar size. This result also indicates that these proteins are, at least, partially biologically connected as a group [61, 62]. The RpoA (DNA-directed RNA polymerase subunit alpha) and Hns (DNA-binding protein H-NS) proteins were the central nodes of two networks that are connected (Fig 4A). RpoA had its abundance increased at 6 h of cultivation of *Salmonella* Enteritidis in the presence of C12-HSL (Fig 4B). Soni et al. [85] showed that the abundance of this protein decreased in late logarithmic phase of growth of *Salmonella* Typhimurium in the presence of AI-2. In addition, these proteins were identified at all the time points evaluated in this study (Fig 4B). Thus, the differential abundance of proteins related to transcription regulation may be responsible for the differences observed in the abundance of other proteins between control and treatment with C12-HSL and throughout the time of cultivation of *Salmonella* (Figs 3 and 4B).

### 3.3. Levels of thiol and proteins related to the oxidation-reduction process are altered by HSL in *Salmonella*

The proteins related to the oxidation-reduction process had their abundance affected by the presence of C12-HSL at all times of cultivation (Fig 3). At 4 h of cultivation with this signaling molecule, a greater number of these proteins had their abundance decreased in comparison to the control (Fig 3). Conversely, their abundance was increased at 6 h of cultivation. This might suggest that cells cultivated during this period in the presence of C12-HSL have greater potential to resist oxidative stress than cells cultivated in the absence of this molecule. The proteins related to the oxidative process can be considered crucial to maintenance of the cellular redox balance, as well as to anticipate resistance to a possible oxidative stress due to excessive production of reactive oxygen/nitrogen species (ROS/RNS) [86–88].

In other bacteria, quorum sensing has been associated to oxidative stress response. For instance, in *P*. *aeruginosa*, the expression of *katA* (catalase) and *sodA* (superoxide dismutase) genes and, concomitantly, the activities of the catalase and superoxide dismutase enzymes were up-regulated by quorum sensing [89]. In addition, Garcia-Contreras et al. [90] showed that resistance of *P*. *aeruginosa* to oxidative stress caused by the addition of hydrogen peroxide (H_2_O_2_) was enhanced due to quorum sensing, increasing the production of catalase and NADPH dehydrogenases. In the study herein, the SodC1 (Superoxide dismutase [Cu-Zn] 1) also had its abundance increased at 4 h of incubation (Log_2_ FC = 11.567), while SodB (Superoxide dismutase [Fe]) protein had its abundance increased at 7 h when in the presence of C12-HSL (Log_2_ FC = 1.417) (Table 3). The cell-cell communication system via BpsIR (homologous to LuxIR) and *N*-octanoyl-homoserine lactone (C8-HSL) also increased resistance to oxidative stress of *B*. *pseudomallei*, as well as the expression of the *dpsA* gene (DNA-binding protein from starved cells) [91]. The Dps is a non-specific DNA-binding protein involved in resistance to oxidative stress and it is an abundant protein in stationary phase of growth in *E*. *coli* [92, 93].

**Fig 5:**
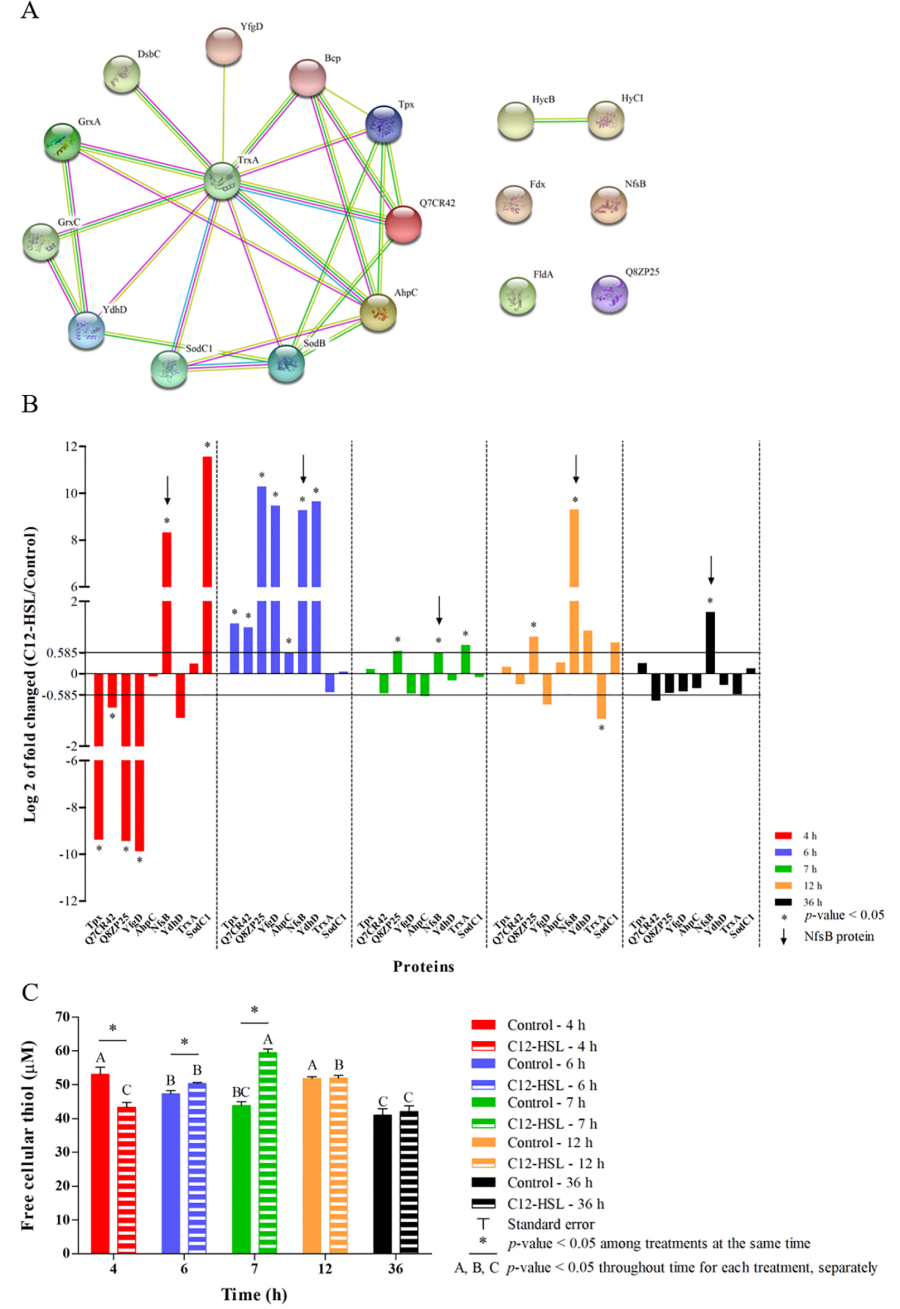
Identified proteins related to the oxidation-reduction process and quantification of free cellular thiol in *Salmonella* Enteritidis PT4 578 anaerobically cultivated in TSB at 37 °C in the presence or absence of C12-HSL. **(A)** The network of interactions among the proteins of oxidation-reduction process and **(B)** the logarithm in base two of fold changed of the proteins that were identified at all time points and in at least one of the treatments, as well as **(C)** the quantification of free cellular thiol in the absence (control) and presence of C12-HSL.

A network of interaction among the proteins of the oxidation-reduction process was generated (Fig 5A). The PPI network showed an average local clustering coefficient of 0.609 and a *p*-value of <1e-16 for enrichment. These results indicate that the interactions showed at medium confidence or better and, the proteins have more interactions among themselves than for a random set of proteins of similar size and also that these proteins are at least partially biologically connected as a group [61, 62].

The predicted network is an efficient tool to identify potential interactions between a large number of proteins identified by proteomics providing a biological meaning for the data. Furthermore, the centrality of proteins in this network is generally associated to the importance of this element to the biological process associated to the network [94, 95]. Interestingly, the TrxA protein (Thioredoxin 1) stands out as being a central node by interacting with most of the proteins used to generate the network (Fig 5A). In these experiments, this protein had its increased abundance at 7 h of cultivation in the presence of C12-HSL, but its abundance was lower at 12 h (Fig 5B). This protein is known to be essential to activate gene transcription of the pathogenicity island 2 (SPI-2) of *Salmonella* Typhimurium and consequently, for resistance during mice infection [96–98].

Thioredoxin is an oxidoreductase that participates in redox reactions by oxidation of its thiol active-sites which are then reduced by NADPH. It also exerts control over the activity of target proteins via reversible thiol-disulfide exchange reactions by the thioredoxin and glutaredoxin systems [88, 99–102]. This protein also has a regulatory mechanism independent of thiol redox activity, which thioredoxin interacts with other proteins and forms a functional complex [100]. Thiol, also known as mercaptan or sulfhydryl,—SH side chain of cysteine is susceptible to reactions with ROS or RNS species, giving rise to a range of post-translational oxidative modifications by thiol proteins, including reversible (intra-protein disulfides, inter-protein disulfides, S-sulfenation, S-nitrosation, S-thiolation, S-sulfhydration, S-sulfenamidation) and non-reversible hyper-oxidized (S-sulfination, S-sulfonation) redox states. In addition, in some cases it can alter the structure and activity of proteins that contain cysteine residues [87, 88, 103, 104].

From nine proteins that were identified at all cultivation times and used for the generation of the network (Fig 5A), eight are or have some relation to thiol proteins such as: Tpx (Probable thiol peroxidase), Q7CR42 (Putative thiol-alkyl hydroperoxide reductase), Q8ZP25 (Putative thiol-disulfide isomerase and thioredoxin), YfgD (Arsenate reductase), AhpC (Alkyl hydroperoxide reductase subunit C), NfsB (Oxygen-insensitive NAD(P)H nitroreductase), YdhD (Glutaredoxin) and TrxA (Thioredoxin 1) proteins. The thiol proteins: Tpx, Q7CR42, Q8ZP25 and YfgD decreased in abundance at 4 h of cultivation of *Salmonella* Enteritidis in the presence of the 50 nM C12-HSL (Fig 5B). On the other hand, at 6 h of cultivation, the Tpx, Q7CR42, Q8ZP25, YfgD, AhpC, NfsB and YdhD proteins increased in abundance, as well as Q8ZP25, NfsB and TrxA proteins at 7 h of cultivation (Fig 5B). In addition, more thiol proteins had their abundances altered at 4, 6 and 7 h of culture, which refer to the logarithmic phase up to the early stationary phase of growth compared to the times of 12 and 36 h where the cells were in stationary phase for a long time (Fig 5B).

The quantification of free cellular thiol showed a correlation with the abundance of thiol proteins at each time and treatment (Fig 5B and 5C). At 4 h of cultivation, a lower level of thiol was observed in the treatment with the quorum sensing molecule as well as a lower abundance of the thiol proteins in comparison to the control. Subsequently, at 6 and 7 h higher levels of thiol were observed in cells cultivated in presence of C12-HSL (Fig 5C and S4 Table). Then, at 12 and 36 h no differences in the levels of thiol were observed, correlating with the number of differentially abundant thiol proteins. In addition, for the same treatment throughout the time of cultivation of *Salmonella*, the levels of thiol increased up to 7 h of cultivation in the presence of C12-HSL and decreased after this time (Fig 5C). On the other hand, in the absence of this AHL, the level of thiol varied throughout time without a trend (Fig 5C). These results showed that quorum sensing alters not only the abundance of thiol proteins but also the levels of thiol, suggesting that resistance to possible oxidative stress can be mediated by the signaling molecule. This is the first time that the relationship between thiol proteins and levels of free cellular thiol with quorum sensing is reported.

Variations in the abundance of thiol proteins and levels of free cellular thiol due to the growth phase of *Salmonella* and the presence of acyl homoserine lactone can be related to changes in the structure of the SdiA protein (LuxR homologue) which could alter its ability to bind DNA and, consequently, activate transcription. On the other hand, the thiol proteins and thiol could prevent structural alterations of the SdiA protein caused by ROS/RNS. This rationale is possible because the cysteine residues (C) and their respective positions (C45, C122, C142, and C232) present in the SdiA protein of *Salmonella* Enteritidis PT4 578 could be susceptible to oxidative stress [105]. The C232 is the main conserved residue among LuxR family proteins [31] and it is involved in the interaction between the Ligand-binding domain (LBD) with DNA-binding domain (DBD) and DBD-DBD [30].

Kafle et al. [106] evaluated all cysteine residues of the LasR protein, a LuxR homologue, of *P*. *aeruginosa* to infer their redox sensitivity and to probe the connection between stress response and the activity of that protein. The C79 residue is important for ligand recognition and folding of this domain which further potentiates DNA binding, but it does not seem to be sensitive to oxidative stress when bound to its native ligand. The C201 and C203 residues in the DBD form a disulfide bond when treated with hydrogen peroxide, and this bond seems to disrupt the DNA binding activity of the transcription factor. Mutagenesis of either of these cysteines leads to expression of a protein that no longer binds DNA. Thus, these authors provided a possible mechanism for oxidative stress response by the cysteine residues of the LasR protein in *P*. *aeruginosa* and indicated that multiple cysteines within the protein can be useful targets for disabling its activity.

The presence of C12-HSL increased the abundance of thiol proteins, such as oxidoreductases, which can change the structure of this AHL and inactivate it. Some oxidoreductases synthesized by *Rhodococcus erythropolis* and *Bacillus megaterium*, as well as by eukaryotic cells, are able to inactivate AHLs by oxidation or reduction of their acyl side chain [107–110]. In fact, this is one of the known mechanisms of quorum quenching [107].

Finally, the NfsB protein (also named NfnB or NfsI) was the only protein that had its abundance increased at all cultivation times in the presence of C12-HSL compared to the control treatment in *Salmonella* (Fig 5B, indicated with a black arrow at each time point). This result suggests that *Salmonella* cultivated in the presence of this quorum sensing molecule can be susceptible to the action of nitrofurans or that C12-HSL has a certain toxicity or mutagenicity activity to the cell. The NfsB protein is a flavin mononucleotide-containing flavoprotein that can use either NADH or NADPH as a source of reducing power in order to reduce the nitro moiety of nitrofurans, yielding biologically inactive end products. This process occurs through a sequence of intermediates, including nitroso and hydroxylamine states, which are assumed to be responsible for toxicity [111, 112]. *E*. *coli* and *Salmonella* Typhimurium containing the *nfsA* and *nfsB* genes are more sensitive to nitrofurans, which are widely used as antimicrobial agents [113, 114]. Carroll et al. [115] also showed that the introduction of plasmids carrying the *nfsA* and *nfsB* genes into *Salmonella* Typhimurium increased sensibility to nitrofuran compounds with mutagenic potential.

Toxicity of AHLs has already been proven in many studies, especially those performed with eukaryotic cells. For instance, Gomi et al. [116] showed that 10 μg/mL of C12-HSL derived from *Chromobacterium violaceum* induced the production of tumor necrosis factor-α (TNF-α) and interleukin-1β (IL-1β) by mouse RAW264.7 cells and IL-8 by human THP-1 cells, while C4, C6, C7, C8, C10 and C14-HSL had no effect. The C12-HSL and C12-oxo-HSL also decreased the levels of putrescine in human epidermal cells (HaCat) and, consequently, decreased the rate of cell proliferation [117]. John et al. [118] showed that *N*-3-oxo-tetradecanoyl-homoserine lactone (3-oxo-C14-HSL) produced by *Acinetobacter baumannii* had a dose-dependent cytotoxic effect on human cervical cancer cells (HeLa), adenocarcinoma human alveolar basal epithelial cells (A549), Dukes’ type C colorectal adenocarcinoma cells (HCT15) and Dukes’ type B colorectal adenocarcinoma cells (SW480), with induction of apoptosis and reduced viability and proliferation of these cells in the presence of AHL. In addition, these authors showed that 3-oxo-C14-HSL was able to decrease the growth of *Staphylococcus aureus*.

It is possible that the NfsB protein can be used as a biomarker for the presence of the AI-1 in *Salmonella*, due to its greater abundance in the presence of this molecule in all the evaluated times. It is noteworthy that nitroreductases homologous to NfsA and NfsB are found in many members of the Enterobacteriaceae family [119].

## 4. Conclusions

**Fig 6:**
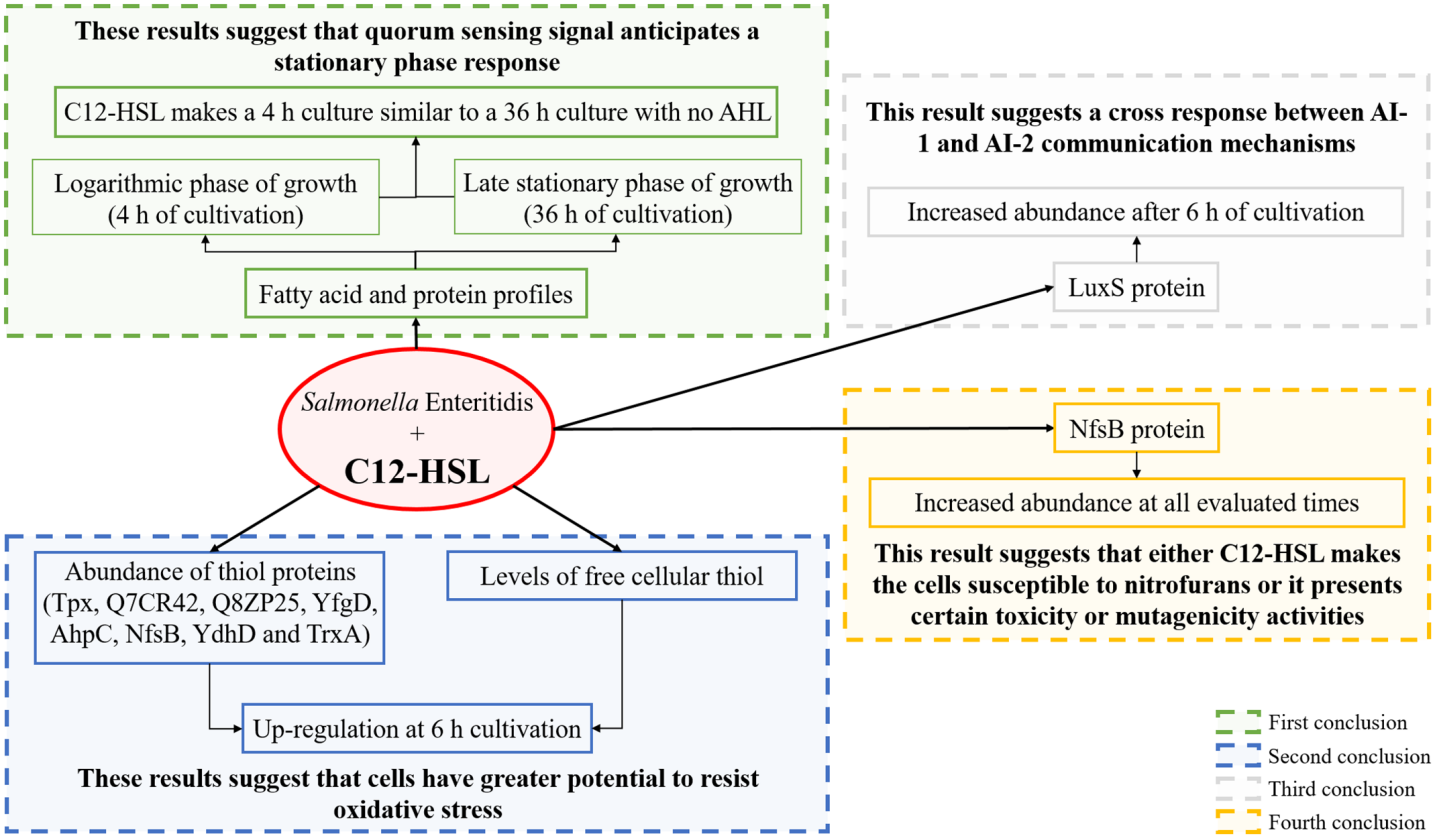
Global response of *Salmonella* to the presence of C12-HSL. The results related to a conclusion.

The fatty acid and protein profiles of *Salmonella* Enteritidis PT4 578 in logarithmic phase of growth at 4 h of cultivation in the presence of C12-HSL were similar to the profiles of cells in late stationary phase at 36 h, suggesting that quorum sensing signal anticipates a stationary phase response (Fig 6). Overall, the fatty acid and protein profiles varied less along the bacterium growth in the presence of AI-1. In addition, the presence of this signaling molecule increased the abundance of thiol proteins and the levels of free cellular thiol after 6 h of cultivation suggesting that the cells may be prepared for a possible oxidative stress (Fig 6). This increase may lead to modifications in the structure of the AHL or SdiA protein which, consequently, could alter its binding affinities to the DNA. More studies are needed in order to confirm this hypothesis.

There was an increased abundance of LuxS protein in presence of C12-HSL which suggests a cross response between the quorum sensing mechanisms mediated by AI-1 and AI-2 in *Salmonella* (Fig 6), while the increased abundance of NfsB protein in this condition suggests that the cells can either be susceptible to the action of nitrofurans or this signaling molecule has a certain toxicity or mutagenicity to the cell (Fig 6). Further studies are needed in order to confirm the hypotheses generated in this work.

## Supporting information

S1 Table Concentration data of the fatty acids of *Salmonella* Enteritidis PT4 578 anaerobically cultivated in TSB at 37 °C in the presence or absence of C12-HSL. (XLSX)

S2 Table Mass spectrometry data by UPLC-Q-Tof of the proteins of *Salmonella* Enteritidis PT4 578 anaerobically cultivated in TSB at 37 °C in the presence or absence of C12-HSL. MM = molecular mass;pI = isoeletric point;TIC = total ion current;Mascot = Mascot software (version 2.4.0; Matrix Science, United Kingdom);Scaffold = Scaffold software (version 4.7.2; Proteome Software, USA).
(XLSX)

S3 Table Organism, gene, protein, molecular mass (MM), isoeletric point (pI), process and function of proteins identified from *Salmonella* Enteritidis PT4 578 anaerobically cultivated in TSB at 37 °C in the presence or absence of C12-HSL. (DOCX)

S4 Table Quantification data of free cellular thiol of *Salmonella* Enteritidis PT4 578 anaerobically cultivated in TSB at 37 °C in the presence or absence of C12-HSL. (DOCX)
