# Supplementary material for: N-dodecanoyl-homoserine lactone influences the levels of thiol and proteins related to oxidation-reduction process in Salmonella
Source: PLoS One. 2018 Oct 10;13(10):e0204673. doi: 10.1371/journal.pone.0204673 (PMC6179229; doi:10.1371/journal.pone.0204673)
Supplement: S3 Table — (DOCX) [file pone.0204673.s003.docx]

**S3 Table. Organism, gene, protein, molecular mass (MM), isoeletric point (pI), process and function of proteins identified from *Salmonella* Enteritidis PT4 578 anaerobically cultivated in TSB at 37 °C in the presence or absence of C12-HSL.**

| **Organism** | **Gene** | **Gene name** | **Protein** | **Protein name** | **MM (KDa)** | **pI** | **Process** | **Function** |
| --- | --- | --- | --- | --- | --- | --- | --- | --- |
| *Salmonella* Typhimurium str. LT2 | *atpH* | STM3868 | ATP synthase subunit delta | Q7CPE5 | 19.514 | 4.89 | Biosynthetic | ATP |
| *Salmonella* Typhimurium str. LT2 | *accB* | STM3379 | Acetyl-CoA carboxylase, BCCP subunit | Q7CPM1 | 16.733 | 4.66 | Biosynthetic | Fatty acid |
| *Salmonella* Typhimurium str. LT2 | *acpP* | STM1196 | Acyl carrier protein | P0A6B1 | 8.634 | 3.98 | Biosynthetic | Fatty acid |
| *Salmonella* Typhimurium str. LT2 | *atpD* | STM3865 | ATP synthase subunit beta | Q7CPE2 | 50.309 | 4.90 | Biosynthetic | Unclassified |
| *Salmonella* Typhimurium str. LT2 | *ftsZ* | STM0133 | Cell division protein FtsZ | Q8ZRU0 | 40.299 | 4.66 | Cell division | FtsZ-dependent cytokinesis |
| *Salmonella* Typhimurium str. LT2 | *zapB* | STM4088 | Cell division protein ZapB | Q8ZKP1 | 9.307 | 4.59 | Cell division | FtsZ-dependent cytokinesis |
| *Salmonella* Typhimurium str. LT2 | *cheZ* | STM1915 | Protein phosphatase CheZ | P07800 | 23.905 | 4.39 | Chemotaxis | Phosphoprotein phosphatase |
| *Salmonella* Typhimurium str. LT2 | *aspA* | STM4326 | Aspartate ammonia-lyase | Q7CPA1 | 52.880 | 5.15 | Metabolic process | Aspartate ammonia-lyase |
| *Salmonella* Typhimurium str. LT2 | *adi* | STM4296 | Arginine decarboxylase | Q8ZKE3 | 84.837 | 5.17 | Metabolic process | Cellular amino acid metabolic |
| *Salmonella* Typhimurium str. LT2 | *aroK* | STM3487 | Shikimate kinase 1 | P63601 | 19.458 | 5.27 | Metabolic process | Cellular amino acid metabolic |
| *Salmonella* Typhimurium str. LT2 | *ridA* | STM4458 | 2-iminobutanoate/2-iminopropanoate deaminase | Q7CP78 | 13.624 | 5.13 | Metabolic process | Cellular amino acid metabolic |
| *Salmonella* Typhimurium str. LT2 | *grcA* | STM2646 | Autonomous glycyl radical cofactor | Q7CQ05 | 14.392 | 5.10 | Metabolic process | Formate C-acetyltransferase |
| *Salmonella* Typhimurium str. LT2 | *pflB* | STM0973 | Pyruvate formate lyase I, induced anaerobically | Q7CQU1 | 85.293 | 5.75 | Metabolic process | Formate C-acetyltransferase |
| *Salmonella* Typhimurium str. LT2 | *gloA* | STM1435 | Lactoylglutathione lyase | P0A1Q2 | 14.869 | 4.90 | Metabolic process | Glutathione metabolic process |
| *Salmonella* Typhimurium str. LT2 | *eno* | STM2952 | Enolase | P64076 | 45.627 | 5.25 | Metabolic process | Glycolytic process |
| *Salmonella* Typhimurium str. LT2 | *gapA* | STM1290 | Glyceraldehyde-3-phosphate dehydrogenase | P0A1P0 | 35.735 | 6.33 | Metabolic process | Glycolytic process |
| *Salmonella* Typhimurium str. LT2 | *pgk* | STM3069 | Phosphoglycerate kinase | P65702 | 41.278 | 5.09 | Metabolic process | Glycolytic process |
| *Salmonella* Typhimurium str. LT2 | *tpiA* | STM4081 | Triosephosphate isomerase | Q8ZKP7 | 26.900 | 5.68 | Metabolic process | Glycolytic process |
| *Salmonella* Typhimurium str. LT2 | *adk* | STM0488 | Adenylate kinase | P0A1V4 | 23.530 | 5.53 | Metabolic process | Nucleotide biosynthetic |
| *Salmonella* Typhimurium str. LT2 | *deoC* | STM4567 | Deoxyribose-phosphate aldolase | Q8ZJV8 | 27.895 | 5.87 | Metabolic process | Nucleotide biosynthetic |
| *Salmonella* Typhimurium str. LT2 | *ppnP* | STM0391 | Pyrimidine/purine nucleoside phosphorylase | Q8ZRE7 | 10.152 | 5.01 | Metabolic process | Nucleotide biosynthetic |
| *Salmonella* Typhimurium str. LT2 | *ackA* | STM2337 | Acetate kinase | P63411 | 43.572 | 5.93 | Metabolic process | Organic acid metabolic |
| *Salmonella* Typhimurium str. LT2 | *rpiA* | STM3063 | Ribose-5-phosphate isomerase A | P66692 | 22.938 | 5.08 | Metabolic process | Pentose-phosphate shunt |
| *Salmonella* Typhimurium str. LT2 | *ribH* | STM0417 | 6,7-dimethyl-8-ribityllumazine synthase | P66038 | 15.998 | 5.10 | Metabolic process | Riboflavin biosynthetic process |
| *Salmonella* Typhimurium str. LT2 | *flgE* | STM1177 | Flagellar hook protein FlgE | P0A1J1 | 42.185 | 4.77 | Motility | Bacterial-type flagellum-dependent cell motility |
| *Salmonella* Typhimurium str. LT2 | *flgL* | STM1184 | Flagellar hook-associated protein 3 | P16326 | 34.154 | 4.83 | Motility | Bacterial-type flagellum-dependent cell motility |
| *Salmonella* Typhimurium str. LT2 | *flgN* | STM1171 | Flagella synthesis protein FlgN | P0A1J7 | 15.979 | 5.34 | Motility | Bacterial-type flagellum-dependent cell motility |
| *Salmonella* Gallinarum str. 287/91 | *fliD* | SG1095 | Flagellar hook-associated protein 2 | B5R7H2 | 49.926 | 5.12 | Motility | Bacterial-type flagellum-dependent cell motility |
| *Salmonella* Gallinarum str. 287/91 | *fljB* | SG1096 | Flagellin | B5R7H3 | 52.950 | 4.90 | Motility | Bacterial-type flagellum-dependent cell motility |

**S3 Table. Continuation.**

| **Organism** | **Gene** | **Gene name** | **Protein** | **Protein name** | **MM (KDa)** | **pI** | **Process** | **Function** |
| --- | --- | --- | --- | --- | --- | --- | --- | --- |
| *Salmonella* Typhimurium str. LT2 | *fdx* | STM2538 | [2FE-2S] ferredoxin | Q7CQ13 | 12.780 | 4.40 | Oxidation-reduction | Electron carrier |
| *Salmonella* Typhimurium str. LT2 | *fldA* | STM0694 | Flavodoxin 1 | Q8ZQX1 | 19.796 | 4.22 | Oxidation-reduction | Electron carrier |
| *Salmonella* Typhimurium str. LT2 | *grxA* | STM0872 | Glutaredoxin 1 | P0A1P8 | 10.089 | 5.63 | Oxidation-reduction | Electron carrier |
| *Salmonella* Typhimurium str. LT2 | *grxC* | STM3702 | Glutaredoxin 3 | Q7CPH7 | 9.301 | 6.70 | Oxidation-reduction | Electron carrier |
| *Salmonella* Typhimurium str. LT2 | *hycB* | STM2852 | Hydrogenase-3, iron-sulfur subunit | Q7CPY1 | 22.610 | 6.63 | Oxidation-reduction | Electron carrier |
| *Salmonella* Typhimurium str. LT2 | *hycI* | STM2845 | Protease involved in processing C-terminal end of HycE | Q8ZMJ3 | 17.097 | 4.00 | Oxidation-reduction | Electron carrier |
| *Salmonella* Typhimurium str. LT2 | *ydhD* | STM1433 | Glutaredoxin | Q7CQK9 | 13.071 | 4.84 | Oxidation-reduction | Electron carrier |
| *Salmonella* Typhimurium str. LT2 | *ahpC* | STM0608 | Alkyl hydroperoxide reductase subunit C | P0A251 | 20.848 | 5.03 | Oxidation-reduction | Oxidoreductase |
| *Salmonella* Typhimurium str. LT2 | *bcp* | STM2491 | Thioredoxin dependent thiol peroxidase | Q7CQ23 | 17.769 | 5.16 | Oxidation-reduction | Oxidoreductase |
| *Salmonella* Typhimurium str. LT2 | *dsbC* | STM3043 | Thiol:disulfide interchange protein DsbC | P55890 | 26.047 | 7.11 | Oxidation-reduction | Oxidoreductase |
| *Salmonella* Typhimurium str. LT2 | *nfsB* | STM0578 | Oxygen-insensitive NAD(P)H nitroreductase | P15888 | 23.997 | 5.40 | Oxidation-reduction | Oxidoreductase |
| *Salmonella* Typhimurium str. LT2 | *sodB* | STM1431 | Superoxide dismutase [Fe] | P0A2F4 | 21.352 | 5.58 | Oxidation-reduction | Oxidoreductase |
| *Salmonella* Typhimurium str. LT2 | *sodC1* | STM1044 | Superoxide dismutase [Cu-Zn] 1 | P0CW86 | 18.529 | 6.48 | Oxidation-reduction | Oxidoreductase |
| *Salmonella* Typhimurium str. LT2 | STM0402 | STM0402 | Putative thiol-alkyl hydroperoxide reductase | Q7CR42 | 22.417 | 5.24 | Oxidation-reduction | Oxidoreductase |
| *Salmonella* Typhimurium str. LT2 | STM1790 | STM1790 | Putative thiol-disulfide isomerase and thioredoxin | Q8ZP25 | 15.052 | 4.69 | Oxidation-reduction | Oxidoreductase |
| *Salmonella* Typhimurium str. LT2 | *tpx* | STM1682 | Probable thiol peroxidase | Q8ZP65 | 18.185 | 4.93 | Oxidation-reduction | Oxidoreductase |
| *Salmonella* Typhimurium str. LT2 | *trxA* | STM3915 | Thioredoxin 1 | P0AA28 | 11.913 | 4.67 | Oxidation-reduction | Oxidoreductase |
| *Salmonella* Typhimurium str. LT2 | *yfgD* | STM2495 | Arsenate reductase | Q8ZN68 | 13.426 | 5.60 | Oxidation-reduction | Oxidoreductase |
| *Salmonella* Enteritidis | *sefA* | sefA | Fimbrial protein | P12061 | 16.524 | 9.65 | Pathogenesis | Cell adhesion |
| *Salmonella* Typhimurium str. LT2 | *phoN* | STM4319 | Non-specific acid phosphatase | P26976 | 18.478 | 9.01 | Pathogenesis | Dephosphorylation |
| *Salmonella* Typhimurium str. LT2 | *lpp1* | STM1377 | Major outer membrane lipoprotein 1 | Q7CQN4 | 8.443 | 9.36 | Pathogenesis | Lipid modification |
| *Salmonella* Typhimurium str. LT2 | *eco* | STM2262 | Ecotin | Q8ZNH4 | 18.320 | 6.59 | Pathogenesis | Serine-type endopeptidase inhibitor |
| *Salmonella* Typhimurium str. LT2 | *fkpA* | STM3453 | Peptidyl-prolyl cis-trans isomerase | Q8ZLL6 | 28.927 | 8.39 | Protein folding | Peptidyl-prolyl cis-trans isomerase |
| *Salmonella* Typhimurium str. LT2 | *ppiB* | STM0536 | Peptidyl-prolyl cis-trans isomerase | Q8XFG8 | 18.243 | 5.52 | Protein folding | Peptidyl-prolyl cis-trans isomerase |
| *Salmonella* Typhimurium str. LT2 | *slyD* | STM3455 | Peptidyl-prolyl cis-trans isomerase | Q8ZLL4 | 21.117 | 4.77 | Protein folding | Peptidyl-prolyl cis-trans isomerase |
| *Salmonella* Typhimurium str. LT2 | *tig* | STM0447 | Trigger factor | P66932 | 48.037 | 4.84 | Protein folding | Peptidyl-prolyl cis-trans isomerase |
| *Salmonella* Typhimurium str. LT2 | *dnaK* | STM0012 | Chaperone protein DnaK | Q56073 | 69.273 | 4.83 | Protein folding | Unfolded protein binding |
| *Salmonella* Typhimurium str. LT2 | *groL* | STM4330 | 60 kDa chaperonin | P0A1D3 | 57.421 | 4.85 | Protein folding | Unfolded protein binding |
| *Salmonella* Typhimurium str. LT2 | *groS* | STM4329 | 10 kDa chaperonin | P0A1D5 | 10.312 | 5.36 | Protein folding | Unfolded protein binding |
| *Salmonella* Typhimurium str. LT2 | *grpE* | STM2681 | Protein GrpE | Q7CPZ4 | 21.827 | 4.69 | Protein folding | Unfolded protein binding |
| *Salmonella* Typhimurium str. LT2 | *ibpA* | STM3809 | Small heat shock protein IbpA | Q7CPF1 | 15.740 | 5.23 | Protein folding | Unfolded protein binding |
| *Salmonella* Typhimurium str. LT2 | *skp* | STM0225 | Chaperone protein Skp | P0A1Z2 | 17.894 | 9.76 | Protein folding | Unfolded protein binding |
| *Salmonella* Typhimurium str. LT2 | *surA* | STM0092 | Chaperone SurA | Q7CR87 | 47.221 | 6.73 | Protein folding | Unfolded protein binding |

**S3 Table. Continuation.**

| **Organism** | **Gene** | **Gene name** | **Protein** | **Protein name** | **MM (KDa)** | **pI** | **Process** | **Function** |
| --- | --- | --- | --- | --- | --- | --- | --- | --- |
| *Salmonella* Typhimurium str. LT2 | *erpA* | STM0204 | Iron-sulfur cluster insertion protein ErpA | Q7CR66 | 12.263 | 4.15 | Protein maturation | Iron-sulfur cluster assembly |
| *Salmonella* Typhimurium str. LT2 | *nfuA* | STM3511 | Fe/S biogenesis protein NfuA | Q8ZLI7 | 21.152 | 4.52 | Protein maturation | Iron-sulfur cluster assembly |
| *Salmonella* Typhimurium str. LT2 | *nifU* | STM2542 | Iron-sulfur cluster assembly scaffold protein IscU | Q7CQ11 | 13.983 | 4.78 | Protein maturation | Iron-sulfur cluster assembly |
| *Salmonella* Typhimurium str. LT2 | *luxS* | STM2817 | S-ribosylhomocysteine lyase | Q9L4T0 | 19.467 | 5.71 | Quorum sensing | S-ribosylhomocysteine lyase |
| *Salmonella* Typhimurium str. LT2 | *ssb* | STM4256 | Single-stranded DNA-binding protein 1 | P0A2F6 | 19.062 | 5.46 | Response to stress | Cellular response to DNA damage stimulus |
| *Salmonella* Typhimurium str. LT2 | *yajQ* | STM0435 | UPF0234 protein YajQ | Q8ZRC9 | 18.308 | 5.60 | Response to stress | Cellular response to DNA damage stimulus |
| *Salmonella* Typhimurium str. LT2 | *uspG* | STM0614 | Universal stress protein G | P67093 | 15.891 | 6.18 | Response to stress | Protein autoadenylation and autophosphorylation |
| *Salmonella* Typhimurium str. LT2 | *ygiW* | STM3176 | Putative outer membrane protein | Q7CPS4 | 13.993 | 5.03 | Response to stress | Protein, cellulose and peptidoglycan binding |
| *Salmonella* Typhimurium str. LT2 | STM1251 | STM1251 | Putative molecular chaperone (Small heat shock protein) | Q8ZPY6 | 17.540 | 5.42 | Response to stress | Unclassified |
| *Salmonella* Typhimurium str. LT2 | *dksA* | STM0186 | RNA polymerase-binding transcription factor DksA | P0A1G5 | 17.733 | 5.06 | Transcription | Amino acid biosynthesis |
| *Salmonella* Typhimurium str. LT2 | *cspC* | STM1837 | Cold shock-like protein CspC | P0A9Y9 | 7.398 | 6.54 | Transcription | Regulation of transcription, DNA-templated |
| *Salmonella* Typhimurium str. LT2 | *cspE* | STM0629 | RNA chaperone, negative regulator of cspA transcription | Q7CQZ5 | 7.447 | 8.09 | Transcription | Regulation of transcription, DNA-templated |
| *Salmonella* Typhimurium str. LT2 | *emrR* | STM2813 | Transcriptional repressor of emrAB operon | Q7CPY9 | 20.752 | 6.07 | Transcription | Regulation of transcription, DNA-templated |
| *Salmonella* Typhimurium str. LT2 | *fur* | STM0693 | Transcriptional repressor of iron-responsive genes (Fur family) (Ferric uptake regulator) | Q7CQY3 | 17.229 | 5.56 | Transcription | Regulation of transcription, DNA-templated |
| *Salmonella* Typhimurium str. LT2 | *greA* | STM3299 | Transcription elongation factor GreA | P64281 | 17.702 | 4.75 | Transcription | Regulation of transcription, DNA-templated |
| *Salmonella* Typhimurium str. LT2 | *hns* | STM1751 | DNA-binding protein H-NS | P0A1S2 | 15.590 | 5.32 | Transcription | Regulation of transcription, DNA-templated |
| *Salmonella* Typhimurium str. LT2 | *hupA* | STM4170 | DNA-binding protein HU-alpha | P0A1R6 | 9.515 | 9.57 | Transcription | Regulation of transcription, DNA-templated |
| *Salmonella* Typhimurium str. LT2 | *phoP* | STM1231 | Virulence transcriptional regulatory protein PhoP | P0DM78 | 25.617 | 5.28 | Transcription | Regulation of transcription, DNA-templated |
| *Salmonella* Typhimurium str. LT2 | *rnk* | STM0616 | Regulator of nucleoside diphosphate kinase | Q7CQZ7 | 15.042 | 4.47 | Transcription | Regulation of transcription, DNA-templated |
| *Salmonella* Typhimurium str. LT2 | *rpoA* | STM3415 | DNA-directed RNA polymerase subunit alpha | P0A7Z7 | 36.717 | 4.98 | Transcription | Regulation of transcription, DNA-templated |
| *Salmonella* Typhimurium str. LT2 | *rpoZ* | STM3741 | DNA-directed RNA polymerase subunit omega | P0A803 | 10.230 | 4.87 | Transcription | Regulation of transcription, DNA-templated |
| *Salmonella* Gallinarum str. 287/91 | SG2019 | SG2019 | DNA-binding protein | B5RBI8 | 14.956 | 5.63 | Transcription | Regulation of transcription, DNA-templated |
| *Salmonella* Typhimurium str. LT2 | *slyA* | STM1444 | Transcriptional regulator SlyA | P40676 | 16.469 | 6.23 | Transcription | Regulation of transcription, DNA-templated |
| *Salmonella* Typhimurium str. LT2 | *stpA* | STM2799 | DNA-binding protein StpA | P0A1S4 | 15.478 | 7.93 | Transcription | Regulation of transcription, DNA-templated |
| *Salmonella* Typhimurium str. LT2 | *ybaB* | STM0485 | Nucleoid-associated protein YbaB | P0A8B8 | 12.064 | 5.01 | Transcription | Regulation of transcription, DNA-templated |
| *Salmonella* Typhimurium str. LT2 | *ydgT* | STM1461 | Transcription modulator YdgT | Q7CQK5 | 8.375 | 6.03 | Transcription | Regulation of transcription, DNA-templated |
| *Salmonella* Typhimurium str. LT2 | *ppa* | STM4414 | Inorganic pyrophosphatase | P65748 | 19.778 | 5.01 | Transcription | RNA degradation |
| *Salmonella* Typhimurium str. LT2 | *rof* | STM0237 | Modulator of Rho-dependent transcription termination | Q8ZRN4 | 9.778 | 4.48 | Transcription | RNA degradation |
| *Salmonella* Typhimurium str. LT2 | *rraA* | STM4089 | Regulator of ribonuclease activity A | P67651 | 17.478 | 4.07 | Transcription | RNA degradation |
| *Salmonella* Typhimurium str. LT2 | *sspB* | STM3341 | Stringent starvation protein B | Q7CPN4 | 18.210 | 4.36 | Transcription | RNA degradation |

**S3 Table. Continuation.**

| **Organism** | **Gene** | **Gene name** | **Protein** | **Protein name** | **MM (KDa)** | **pI** | **Process** | **Function** |
| --- | --- | --- | --- | --- | --- | --- | --- | --- |
| *Salmonella* Typhimurium str. LT2 | *def* | STM3406 | Peptide deformylase | Q8ZLM7 | 19.384 | 5.02 | Translation | Peptide deformylase |
| *Salmonella* Typhimurium str. LT2 | *rrf or frr* | STM0219 | Ribosome recycling factor | P66738 | 20.600 | 7.77 | Translation | Ribosome binding |
| *Salmonella* Typhimurium str. LT2 | *sra* | STM1565 | Stationary-phase-induced ribosome-associated protein | Q7CQJ0 | 5.366 | 11.33 | Translation | Ribosome binding |
| *Salmonella* Typhimurium str. LT2 | *yfiA* | STM2665 | Ribosome associated factor | Q7CQ00 | 12.645 | 6.59 | Translation | Ribosome binding |
| *Salmonella* Typhimurium str. LT2 | *infA* | STM0953 | Translation initiation factor IF-1 | P69226 | 8.244 | 9.22 | Translation | RNA binding |
| *Salmonella* Typhimurium str. LT2 | *infC* | STM1334 | Translation initiation factor IF-3 | P33321 | 20.638 | 9.54 | Translation | RNA binding |
| *Salmonella* Typhimurium str. LT2 | *rplA* | STM4150 | 50S ribosomal protein L1 | P0A2A3 | 24.713 | 9.64 | Translation | Structural constituent of ribosome |
| *Salmonella* Typhimurium str. LT2 | *rplC* | STM3440 | 50S ribosomal protein L3 | P60446 | 22.291 | 9.79 | Translation | Structural constituent of ribosome |
| *Salmonella* Typhimurium str. LT2 | *rplF* | STM3425 | 50S ribosomal protein L6 | P66313 | 18.905 | 9.71 | Translation | Structural constituent of ribosome |
| *Salmonella* Typhimurium str. LT2 | *rplI* | STM4394 | 50S ribosomal protein L9 | Q8ZK80 | 15.774 | 6.75 | Translation | Structural constituent of ribosome |
| *Salmonella* Typhimurium str. LT2 | *rplJ* | STM4151 | 50S ribosomal protein L10 | P0A297 | 17.846 | 9.04 | Translation | Structural constituent of ribosome |
| *Salmonella* Typhimurium str. LT2 | *rplK* | STM4149 | 50S ribosomal protein L11 | P0A7K0 | 14.923 | 9.64 | Translation | Structural constituent of ribosome |
| *Salmonella* Typhimurium str. LT2 | *rplL* | STM4152 | 50S ribosomal protein L7/L12 | P0A299 | 12.291 | 4.60 | Translation | Structural constituent of ribosome |
| *Salmonella* Typhimurium str. LT2 | *rplO* | STM3421 | 50S ribosomal protein L15 | P66073 | 14.957 | 11.18 | Translation | Structural constituent of ribosome |
| *Salmonella* Typhimurium str. LT2 | *rplQ* | STM3414 | 50S ribosomal protein L17 | Q7CPL7 | 14.443 | 11.05 | Translation | Structural constituent of ribosome |
| *Salmonella* Typhimurium str. LT2 | *rplR* | STM3424 | 50S ribosomal protein L18 | Q7CPL6 | 12.762 | 10.46 | Translation | Structural constituent of ribosome |
| *Salmonella* Typhimurium str. LT2 | *rplS* | STM2673 | 50S ribosomal protein L19 | P0A2A1 | 13.122 | 10.80 | Translation | Structural constituent of ribosome |
| *Salmonella* Typhimurium str. LT2 | *rplX* | STM3429 | 50S ribosomal protein L24 | P60626 | 11.309 | 10.21 | Translation | Structural constituent of ribosome |
| *Salmonella* Typhimurium str. LT2 | *rplY* | STM2224 | 50S ribosomal protein L25 | Q7CQ71 | 10.535 | 9.52 | Translation | Structural constituent of ribosome |
| *Salmonella* Typhimurium str. LT2 | *rpmB* | STM3728 | 50S ribosomal protein L28 | P0A2A5 | 9.102 | 11.42 | Translation | Structural constituent of ribosome |
| *Salmonella* Typhimurium str. LT2 | *rpmC* | STM3432 | 50S ribosomal protein L29 | P66170 | 7.256 | 9.98 | Translation | Structural constituent of ribosome |
| *Salmonella* Typhimurium str. LT2 | *rpmD* | STM3422 | 50S ribosomal protein L30 | P0A2A7 | 6.510 | 10.96 | Translation | Structural constituent of ribosome |
| *Salmonella* Typhimurium str. LT2 | *rpmE* | STM4096 | 50S ribosomal protein L31 | P66191 | 7.942 | 9.51 | Translation | Structural constituent of ribosome |
| *Salmonella* Typhimurium str. LT2 | *rpmG* | STM3727 | 50S ribosomal protein L33 | P0A7P2 | 6.368 | 10.25 | Translation | Structural constituent of ribosome |
| *Salmonella* Typhimurium str. LT2 | *rpmH* | STM3839 | 50S ribosomal protein L34 | P0A7P8 | 5.377 | 13.00 | Translation | Structural constituent of ribosome |
| *Salmonella* Typhimurium str. LT2 | *rpsA* | STM0981 | 30S ribosomal protein S1 | Q7CQT9 | 61.250 | 4.89 | Translation | Structural constituent of ribosome |
| *Salmonella* Typhimurium str. LT2 | *rpsB* | STM0216 | 30S ribosomal protein S2 | P66541 | 26.799 | 6.62 | Translation | Structural constituent of ribosome |
| *Salmonella* Typhimurium str. LT2 | *rpsD* | STM3416 | 30S ribosomal protein S4 | O54297 | 23.528 | 10.02 | Translation | Structural constituent of ribosome |
| *Salmonella* Typhimurium str. LT2 | *rpsE* | STM3423 | 30S ribosomal protein S5 | P0A7W4 | 17.592 | 10.11 | Translation | Structural constituent of ribosome |
| *Salmonella* Typhimurium str. LT2 | *rpsF* | STM4391 | 30S ribosomal protein S6 | P66593 | 15.163 | 5.26 | Translation | Structural constituent of ribosome |
| *Salmonella* Typhimurium str. LT2 | *rpsG* | STM3447 | 30S ribosomal protein S7 | P0A2B3 | 17.579 | 10.30 | Translation | Structural constituent of ribosome |
| *Salmonella* Typhimurium str. LT2 | *rpsH* | STM3426 | 30S ribosomal protein S8 | P0A7X0 | 14.175 | 9.44 | Translation | Structural constituent of ribosome |
| *Salmonella* Typhimurium str. LT2 | *rpsJ* | STM3441 | 30S ribosomal protein S10 | P67904 | 11.759 | 9.85 | Translation | Structural constituent of ribosome |

**S3 Table. Continuation.**

| **Organism** | **Gene** | **Gene name** | **Protein** | **Protein name** | **MM (KDa)** | **pI** | **Process** | **Function** |
| --- | --- | --- | --- | --- | --- | --- | --- | --- |
| *Salmonella* Typhimurium str. LT2 | *rpsK* | STM3417 | 30S ribosomal protein S11 | O54296 | 13.936 | 11.33 | Translation | Structural constituent of ribosome |
| *Salmonella* Typhimurium str. LT2 | *rpsM* | STM3418 | 30S ribosomal protein S13 | Q8ZLM1 | 13.210 | 10.78 | Translation | Structural constituent of ribosome |
| *Salmonella* Typhimurium str. LT2 | *rpsN* | STM3427 | 30S ribosomal protein S14 | P66409 | 11.658 | 11.16 | Translation | Structural constituent of ribosome |
| *Salmonella* Typhimurium str. LT2 | *rpsR* | STM4393 | 30S ribosomal protein S18 | Q8ZK81 | 9.123 | 10.78 | Translation | Structural constituent of ribosome |
| *Salmonella* Typhimurium str. LT2 | *rpsS* | STM3436 | 30S ribosomal protein S19 | P66491 | 10.410 | 10.52 | Translation | Structural constituent of ribosome |
| *Salmonella* Typhimurium str. LT2 | *rpsT* | STM0043 | 30S ribosomal protein S20 | P0A2B1 | 9.649 | 11.18 | Translation | Structural constituent of ribosome |
| *Salmonella* Typhimurium str. LT2 | *efp* | STM4334 | Elongation factor P | P64036 | 20.667 | 4.90 | Translation | Translation elongation factor |
| *Salmonella* Typhimurium str. LT2 | *tsf* | STM0217 | Elongation factor Ts | P64052 | 30.453 | 5.13 | Translation | Translation elongation factor |
| *Salmonella* Typhimurium str. LT2 | *tufA* | STM3445 | Elongation factor Tu | P0A1H5 | 43.427 | 5.30 | Translation | Translation elongation factor |
| *Salmonella* Typhimurium str. LT2 | *hisJ* | STM2354 | Histidine-binding periplasmic protein | P02910 | 28.476 | 6.03 | Transport | Amino acid transport |
| *Salmonella* Typhimurium str. LT2 | *crr* | STM2433 | PTS system glucose-specific EIIA component | P0A283 | 18.236 | 4.73 | Transport | Carbohydrate transport |
| *Salmonella* Typhimurium str. LT2 | *fruB* | STM2206 | Multiphosphoryl transfer protein | P17127 | 39.569 | 4.87 | Transport | Carbohydrate transport |
| *Salmonella* Typhimurium str. LT2 | *ptsH* | STM2431 | Phosphocarrier protein HPr | P0AA07 | 9.114 | 5.65 | Transport | Carbohydrate transport |
| *Salmonella* Typhimurium str. LT2 | *glnH* | STM0830 | Glutamine high-affinity transporter | Q7CQW0 | 27.245 | 8.44 | Transport | Ionotropic glutamate receptor |
| *Salmonella* Typhimurium str. LT2 | *ftn* | STM1935 | Ferritin | Q8ZNU4 | 19.324 | 4.89 | Transport | Iron ion transport |
| *Salmonella* Typhimurium str. LT2 | *secB* | STM3701 | Protein-export protein SecB | Q7CPH8 | 17.462 | 4.26 | Transport | Protein transport |
| *Salmonella* Typhimurium str. LT2 | *ompA* | STM1070 | Outer membrane protein A | P02936 | 37.606 | 5.60 | Transport | Structural molecule |
| *Salmonella* Typhimurium str. LT2 | *tolC* | STM3186 | Outer membrane channel | Q8ZLZ4 | 53.653 | 5.42 | Transport | Structural molecule |
| *Salmonella* Enteritidis | ABA47_0691 | ABA47_0691 | BssS protein family | A0A1V9AFN8 | 9.256 | 4.66 | Unclassified | Unclassified |
| *Salmonella* sp. str. HMSC13B08 | HMPREF  3126_08675 | HMPREF  3126_08675 | Uncharacterized protein | A0A1F2JWR0 | - | - | Unclassified | Unclassified |
| *Salmonella* Enteritidis | R567_04560 | R567_04560 | Uncharacterized protein | A0A1R2IBX3 | 8.597 | 10.29 | Unclassified | Unclassified |
| *Salmonella* Gallinarum str. 287/91 | SG1997 | SG1997 | Uncharacterized protein | B5RBG9 | 8.584 | 10.37 | Unclassified | Unclassified |
| *Salmonella* Typhimurium str. LT2 | STM1249 | STM1249 | Putative periplasmic protein | Q8ZPY8 | 12.681 | 6.21 | Unclassified | Unclassified |
| *Salmonella* Typhimurium str. LT2 | *yaeP* | STM0238 | UPF0253 protein YaeP | P67551 | 7.266 | 4.66 | Unclassified | Unclassified |
| *Salmonella* Typhimurium str. LT2 | *yccJ* | STM1118 | Putative cytoplasmic protein | Q8ZQ41 | 8.684 | 4.74 | Unclassified | Unclassified |
| *Salmonella* Typhimurium str. LT2 | *ycfF* | STM1205 | Uncharacterized protein | Q7CQR0 | 13.261 | 5.74 | Unclassified | Unclassified |
| *Salmonella* Typhimurium str. LT2 | *ydfZ* | STM1509 | Putative cytoplasmic protein | Q7CQJ6 | 7.275 | 9.09 | Unclassified | Unclassified |
| *Salmonella* Typhimurium str. LT2 | *yecF* | STM1949 | Putative cytoplasmic protein | Q7CQB7 | 8.234 | 4.85 | Unclassified | Unclassified |
| *Salmonella* Typhimurium str. LT2 | *yeeX* | STM2059 | UPF0265 protein YeeX | P67605 | 13.065 | 9.10 | Unclassified | Unclassified |
| *Salmonella* Typhimurium str. LT2 | *yfcZ* | STM2390 | Putative cytoplasmic protein | Q7CQ33 | 10.281 | 4.13 | Unclassified | Unclassified |
| *Salmonella* Typhimurium str. LT2 | *yjbR* | STM4251 | Putative cytoplasmic protein | Q8ZKH3 | 13.377 | 6.05 | Unclassified | Unclassified |
| *Salmonella* Salamae | - | - | Uncharacterized protein | I3W485 | 38.615 | 9.29 | Unclassified | Unclassified |

MM = molecular mass;

pI = isoeletric point.
