# Supplementary material for: N-dodecanoyl-homoserine lactone influences the levels of thiol and proteins related to oxidation-reduction process in Salmonella
Source: PLoS One. 2018 Oct 10;13(10):e0204673. doi: 10.1371/journal.pone.0204673 (PMC6179229; doi:10.1371/journal.pone.0204673)
Supplement: S4 Table — (DOCX) [file pone.0204673.s004.docx]

**S4 Table. Quantification data of free cellular thiol of *Salmonella* Enteritidis PT4 578 anaerobically cultivated in TSB at 37 °C in the presence or absence of C12-HSL.**

| **Time (h)** | **Free cellular thiol (µM)** | | | | | | | | |
| --- | --- | --- | --- | --- | --- | --- | --- | --- | --- |
|  | **Control** | | | |  | **C12-HSL** | | | |
|  | **Quadruplicate** | | | |  | **Quadruplicate** | | | |
|  | **1** | **2** | **3** | **4** |  | **1** | **2** | **3** | **4** |
| 4 | 55.83 | 53.53 | 51.32 | 51.41 |  | 44.57 | 41.59 | 44.39 | 42.88 |
| 6 | 48.52 | 46.73 | 47.53 | 46.35 |  | 50.34 | 49.94 | 50.80 | 50.11 |
| 7 | 43.58 | 42.87 | 45.54 | 43.55 |  | 59.76 | 58.08 | 59.85 | 60.44 |
| 12 | 52.29 | 52.09 | 51.60 | 51.25 |  | 51.47 | 52.90 | 52.21 | 51.12 |
| 36 | 40.16 | 39.65 | 40.22 | 43.88 |  | 41.22 | 40.23 | 43.12 | 43.81 |
